# Extracting Tag Hierarchies

**DOI:** 10.1371/journal.pone.0084133

**Published:** 2013-12-31

**Authors:** Gergely Tibély, Péter Pollner, Tamás Vicsek, Gergely Palla

**Affiliations:** 1 Dept. of Biological Physics, Eötvös University, Budapest, Hungary; 2 Statistical and Biological Physics Research Group of HAS, Budapest, Hungary; 3 Eötvös University, Regional Knowledge Centre, Székesfehervár, Hungary; Semmelweis University, Hungary

## Abstract

Tagging items with descriptive annotations or keywords is a very natural way to compress and highlight information about the properties of the given entity. Over the years several methods have been proposed for extracting a hierarchy between the tags for systems with a "flat", egalitarian organization of the tags, which is very common when the tags correspond to free words given by numerous independent people. Here we present a complete framework for automated tag hierarchy extraction based on tag occurrence statistics. Along with proposing new algorithms, we are also introducing different quality measures enabling the detailed comparison of competing approaches from different aspects. Furthermore, we set up a synthetic, computer generated benchmark providing a versatile tool for testing, with a couple of tunable parameters capable of generating a wide range of test beds. Beside the computer generated input we also use real data in our studies, including a biological example with a pre-defined hierarchy between the tags. The encouraging similarity between the pre-defined and reconstructed hierarchy, as well as the seemingly meaningful hierarchies obtained for other real systems indicate that tag hierarchy extraction is a very promising direction for further research with a great potential for practical applications. Tags have become very prevalent nowadays in various online platforms ranging from blogs through scientific publications to protein databases. Furthermore, tagging systems dedicated for voluntary tagging of photos, films, books, etc. with free words are also becoming popular. The emerging large collections of tags associated with different objects are often referred to as folksonomies, highlighting their collaborative origin and the “flat” organization of the tags opposed to traditional hierarchical categorization. Adding a tag hierarchy corresponding to a given folksonomy can very effectively help narrowing or broadening the scope of search. Moreover, recommendation systems could also benefit from a tag hierarchy.

## Introduction

The appearance of tags in various online contents have become very common, e.g., tags indicate the topic of news-portal feeds and blog post, the genre of films or music records on file sharing portals, or the kind of goods offered in Web stores. By summarizing the most important properties of an entity in only a few words we “compress” information and provide a rough description of the given entity which can be processed very rapidly, (e.g., the user can decide whether the given post is of interest or not without actually reading it). The usage of tags, keywords, categories, etc., for helping the search and browsing amongst a large number of objects is a general idea that has been around for a long time in, e.g., scientific publications, library classification systems and biological classification. However, in the former examples the tagging (categorization) of the involved entities is hierarchical, with a set of narrower or broader categories building up a tree-like structure composed of “is a subcategory of” type relations. In contrast, the nature of tags appearing in online systems is rather different: they can usually correspond to any free word relevant to the tagged item, and they are almost never organized into a pre-defined hierarchy of categories and sub-categories [1]–[3]. Moreover, in some cases they originate from extensive collaboration as, e.g., in tagging systems like Flickr, CiteUlike or Delicious [4]–[6], where unlimited number of users can tag photos, Web pages, etc., with free words. The arising set of free tags and associated objects are usually referred to as folksonomies, for emphasizing their collaborative nature. Since each tagging action is forming a new user-tag-object triple in these systems, their natural representation is given by tri-partite graphs, or in a more general framework by hypergraphs [5], [7]–[9], where the hyperedges connect more than two nodes together.

One of the very interesting challenges related to systems with free tagging is extracting a hierarchy between the appearing tags. Although most tagging systems are intrinsically egalitarian, the way users think about objects presumably has some built in hierarchy, e.g., “poodle” is usually considered as a special case of “dog”. By revealing this sort of hierarchy from, e.g., tag co-occurrence statistics, we can significantly help broadening or narrowing the scope of search in the system, give recommendation about yet unvisited objects to the user [11], [12], or help the categorization of newly appearing objects. Beside the high relevance for practical applications, this problem is interesting also from the theoretical point of view, as marked by several alternative approaches proposed in the recent years. P. Heymann and H. Garcia-Molina introduced a tag hierarchy extracting algorithm based on analyzing node centralities in a co-occurrence network between the tags [13], where connections between tags indicate the appearance of the tags on the same objects simultaneously and link weights correspond to the frequency of co-occurrences. Another interesting approach was outlined by A. Plangprasopchok and K. Lerman [14], [15], which can be applied to systems where users may define a shallow hierarchy for their own tags, and by agglomerating these shallow hierarchies we gain a global hierarchy between the tags. Further notable algorithms were given by P. Schmitz [16], using a probabilistic model and C. Van Damme et al. [17], integrating information from as many sources as possible.

In this paper we introduce a detailed framework for tag hierarchy extraction. Our intended main contributions to this field here are represented by the development of a synthetic, computer generated benchmark system, and the introduction of quality measures for extracted hierarchies. The basic idea of the benchmark system is to simulate the tagging of virtual objects with tags based on a pre-defined input hierarchy between the tags. When applying a hierarchy extraction algorithm to the generated data, the obtained tag hierarchy can be compared to the original tag hierarchy used in the simulation. By changing the parameters of the simulations we can test various properties of the tag hierarchy extracting algorithm in a controlled way. The different quality measures we introduce can be used to evaluate the results of a tag hierarchy extracting algorithm when the exact hierarchy between the tags is also known, (as, e.g., in case of the synthetic benchmark). Furthermore, we also develop new hierarchy extraction methods, which are competitive with the state of the art current methods.

These methods are tested on both the synthetic benchmark and on a couple of real systems as well. One of our data set contains proteins tagged with protein functions, where the extracted tag hierarchy can be compared to the protein function hierarchy of the Genome Ontology. The other real systems included in our study are given by tagged photos from the photo sharing platform Flickr and tagged movies from the Internet Movie Database (IMDb). In these cases, pre-defined “exact” tag hierarchies are not given, therefore, the outcomes of our hierarchy extraction algorithm can be evaluated only by visual inspection of smaller subgraphs in the obtained hierarchies. Luckily, as the tags correspond to English words in these systems, we can still get a good impression whether the obtained hierarchies are meaningful or not.

Our tag hierarchy extraction methods are rooted in complex network theory. In the last 15 years the network approach has become an ubiquitous tool for analyzing complex systems [18], [19]. Networks corresponding to realistic systems can be highly non-trivial, characterized by a low average distance combined with a high average clustering coefficient [20], anomalous degree distributions [21], [22] and an intricate modular structure [23]–[25]. The appearance of node tags is very common in e.g., biological networks,[26]–[31], where they usually refer to the biological function of the units represented by the nodes (proteins, genes, etc.). Node features are also fundamental ingredients in the so-called co-evolving network models, where the evolution of the network topology affects the node properties and vice versa [32]–[37]. Meanwhile, hierarchical organization is yet another very relevant concept in network theory [38]–[44]. As networks provide a sort of “backbone” description for systems in biology, physics, chemistry, sociology, etc., whenever the related system is hierarchical, naturally, the given network is likely to preserve this aspect to some degree. This is supported by several recent studies, focusing on the dominant-subordinate hierarchy among crayfish [45], the leader-follower network of pigeon flocks [46], the rhesus macaque kingdoms [47], the structure of the transcriptional regulatory network of Escherichia coli [48], and on a wide range of social [49]–[51] and technological networks [41].

The two network based tag hierarchy extraction methods presented in this paper are both relying on the weighted network between the tags based on co-occurrence statistics. For the majority of the tags, the direct ancestor in the hierarchy is actually chosen from its neighbors in the network according to various delicate measures.

## Results

### Algorithms

The reason for including both algorithm A and algorithm B in the paper is that algorithm A “wins” on the protein function data set, while algorithm B is better on the computer generated benchmarks and also seems to produce even more meaningful results in case of Flickr and IMDb. We made free implementation of both methods available at (http://hiertags.elte.hu).

#### Algorithm A

The first stage corresponds to defining weighted links between the tags. Probably the most natural choice is given by the number of co-occurrences, (the number of objects tagged simultaneously by the given two tags). Since we are aiming at a directed network, (in which links are pointing from tags higher in the hierarchy towards descendants lower in the hierarchy), in this initial stage we actually assume two separate links pointing in the opposite direction for every pair of co-occurring tags, (with both links having the same weight).

In the next step we prune the network by throwing away a part of the links. Instead applying a global threshold, for each tag 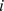 we remove incoming links with a weight smaller than 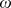 fraction of the weight of the strongest incoming link on 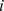. According to our tests on the protein function data set, the quality of the results was only slightly effected by changing 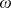. (Our quality measures and the description of the data sets are given in forthcoming sections). Nevertheless, an optimal plateau was observed in the quality as a function of 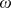 between 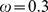 and 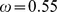, as discussed in details Sect.S1.1.2 in File S1. Thus, in the rest of the paper we show results obtained at 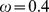.

After the complete link removal process has been finished, the direct ancestor of tag 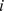 is chosen from the remaining in-neighbors as follows. We calculate the 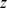-score for the co-occurrence with each in-neighbor individually, given by the difference between the number of observed co-occurrences and the number of expected co-occurrences at random, scaled by the standard deviation, (based on the tag frequencies, more details on the 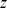-score are given in Methods). The in-neighbor 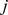 with the highest 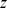-score is usually identified as the direct ancestor, and all other incoming links are deleted on 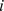. However, there is a very important exception to this rule: in case the 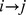 link “survived” when thresholding the incoming links on 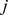. This means that 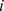 happens to be also a candidate for the ancestor of 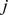, and actually the two tags are more likely to be siblings. In this scenario we go down the list of remaining in-neighbors of 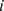 in the order of the 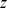-score, until we find a candidate 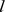 for which the link 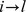 was already deleted, and identify 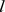 as the ancestor of 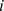. In case no such in-neighbor can be found, 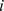 becomes a local root, with temporally no incoming links.

In the last phase of the algorithm we first choose a global root from the local ones according to the maximum entropy of their incoming link weight distribution: if the incoming link weights on 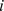 are given by 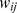 with 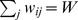, then entropy can be written as 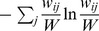. The reasoning behind this choice is that a large entropy usually corresponds to a large number of direct descendants with more or less uniform weight distribution. After the global root has been chosen, we go through the list of local roots in the order of their entropy, and link them under their partner with which they co-occur most frequently. (To avoid the formation of loops, we choose only from co-occurring partners located in another subtree).

The result of the algorithm is a directed tree, since we assign one direct ancestor to every tag during the process, (except the global root), and we do not allow loops. The complexity of the algorithm can be estimated as 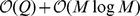, where 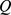 denotes the number of objects, and 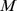 stands for the number of links in the co-occurrence network between the tags. (The details and the pseudo code of the algorithm are given in Sect.S1.1.1 in the File S1).

#### Algorithm B

In case of algorithm B the weight of the links in the network between the tags is the same as in algorithm A, namely the number of objects the tags co-occurred on. However, instead of parallel directed links pointing in the opposite direction, here we consider only single undirected links. Similarly to algorithm A, in the second phase we remove a part of the links from the network. However, in this case we use the 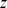-score between connected pairs as a threshold, i.e., if the 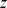-score is below 10, the given link is thrown away. (The optimal value for the 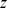-score threshold was set based on experiments on our synthetic benchmark, as detailed in Sect.S1.2.2 in File S1.) There is one exception to the above rule of thresholding: if a tag appears on more than half of the objects of the other tag, then the corresponding link is kept even if the 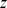-score is low.

Next, the eigenvector centrality is calculated for the tags based on the weighted undirected network remaining after the thresholding, and the tags are sorted according to their centrality value. The hierarchy is built from bottom up: starting from the tag with the lowest eigenvector centrality we choose the direct ancestor of the given tag from its remaining neighbors according to a couple of simple rules. First of all, the ancestor must have a higher centrality. The reasoning behind this is that the eigenvector centrality is analogous to PageRank. Thus, the centrality of a tag is high if it is connected to many other high centrality tags, and therefore, higher centrality values are likely to appear on more frequent and more general tags.

In case the tag 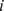 has more than one remaining neighbor with a higher centrality value, we choose the candidate which is the most related to 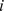 and the set of tags already classified as a descendant of 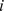. This is implemented by aggregating the 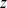-score between the given candidate and the tags in the branch starting from 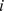, (including 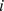 as well), and selecting according to the highest aggregated 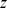-score value. We note that this is a unique feature of the algorithm: by aggregating over the descendants of 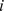 we are using more information compared to simple similarity measures, and hence, are more likely to choose the most related candidate as the parent of 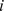.

Since we iterate over the tags in reverse order according to their centrality value, and ancestors have always higher centralities compared to their descendants, no loops are formed during the procedure. The complexity of the method can be estimated as 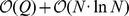, where 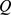 stands for the number of objects and 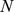 denotes the number of different tags. (The details and the pseudo code of the algorithm are given in Sect.S1.2.1 in the File S1).

### Measuring the quality of the extracted tag hierarchy

#### Simple quality measures

Before actually discussing the results given by tag hierarchy extracting methods in different systems, we need to specify a couple of measures for quantifying the quality of the obtained hierarchies. The natural representation of a hierarchy is given by a directed acyclic graph (DAG), in which links are pointing from nodes at higher level in the hierarchy towards related other nodes lower in the hierarchy. If the exact tag hierarchy is known, the problem is mapped onto measuring the similarity between the DAG obtained from the tag hierarchy extraction method, the “reconstructed” graph, 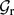 and the exact DAG, 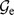.

A simple and natural idea is taking the ratio of exactly matching links in 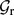, denoted by 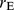, as a primary indicator. In case 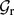 has only a single connected component, 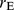 is simply given by the number of links also present in 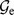, divided by the total number of links in 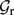, denoted by 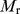. However, if 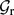 contains only a few links with a vast number of isolated nodes, this sort of normalization can lead to a unrealistically high 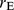 value, in case the links happen to be exactly matching. Thus, in the general case we normalize the number of exactly matching links by 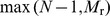, where 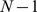 corresponds to the number of links needed for creating a tree between the 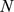 tags.

In a more tolerant approach we may also accept links between more distant ancestor descendant pairs according to the exact hierarchy, (e.g., links pointing from “grandparents” to “grandchildren”). Beside the ratio of acceptable links, 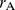, we can measure the ratio of links between unrelated tags, 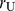 as well, (these are pairs which are not connected by any directed path in 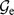), and also the ratio of “inverted” links, 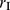, pointing in the opposite direction compared to 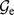, or connecting more distant ancestor descendant pairs in the wrong direction. Furthermore, when 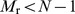, the ratio of missing links from 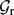, denoted by 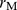, is another important indicator of the effectiveness of the algorithm. (If 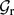 is composed of only a single component, 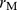 is 0 by definition.) Similarly to 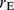, all quality indicators introduced so far are normalized by 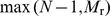. These measures are not completely independent of each other, i.e., the ratio of acceptable links is always larger than or equal to the ratio of exactly matching links, 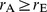, and also 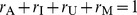.

#### Normalized mutual information between hierarchies

A somewhat more elaborate approach to measuring the quality of the reconstructed hierarchy can be given by the normalized mutual information, (NMI), introduced originally in information theory for measuring the mutual dependence of two random variables [52], [53]. (The definition of the NMI in general is given in Methods). A very important application of the NMI is related to the problem of comparing different partitioning of the same graph into communities [54], [55]. The advantage of the NMI approach when comparing hierarchies is that the resulting similarity measure is sensitive not only to the amount of non-matching links, but also to the position of these links in the hierarchies. In other words, the change in the similarity is different for rewiring a link pointing to a leaf and for rewiring a link higher in the hierarchy.

When judging the similarity between two hierarchies, a natural idea is to compare the sets of descendants for each tag in the corresponding DAGs. E.g., if the set of descendants of tag 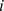 is 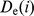 in the exact hierarchy and 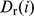 in the reconstructed one, then the number of tags in the intersection of these two sets is given by 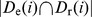. Roughly speaking, the higher the value of this quantity over all tags, the higher is the similarity between the two hierarchies. To build a similarity measure from this concept in the spirit of the NMI, first we define 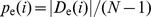 as the probability for picking a tag from the descendants of 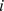 at random in the exact hierarchy, where 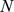 denotes the total number of tags in 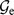. (Since the tag 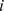 is not included in 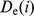, the possible maximum value for 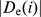 is 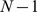). Similarly, the probability for choosing a tag from the descendants of 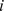 at random in 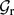 is given by 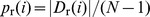, while the probability for picking a tag from the intersection between the descendants of 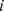 in the two hierarchies can be written as 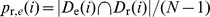. Based on this, the NMI between the exact- and reconstructed hierarchies can be formulated as 
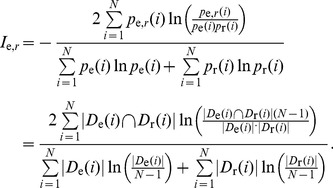
(1)


This measure is 1 if and only 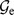 and 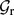 are identical, and is 0 if the intersections between the corresponding branches in the two hierarchies is of the same magnitude as we would expect at random, or in other words, if 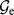 and 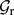 are independent. The similarity defined in the above way is very closely related to the NMI used in community detection [54], [55], the analogy between the two quantities can be made explicit by an appropriate mapping from the hierarchy between the tags to a partitioning of the tags, (further details are given in Sect.S2.1 in File S1).

**Figure 1:**
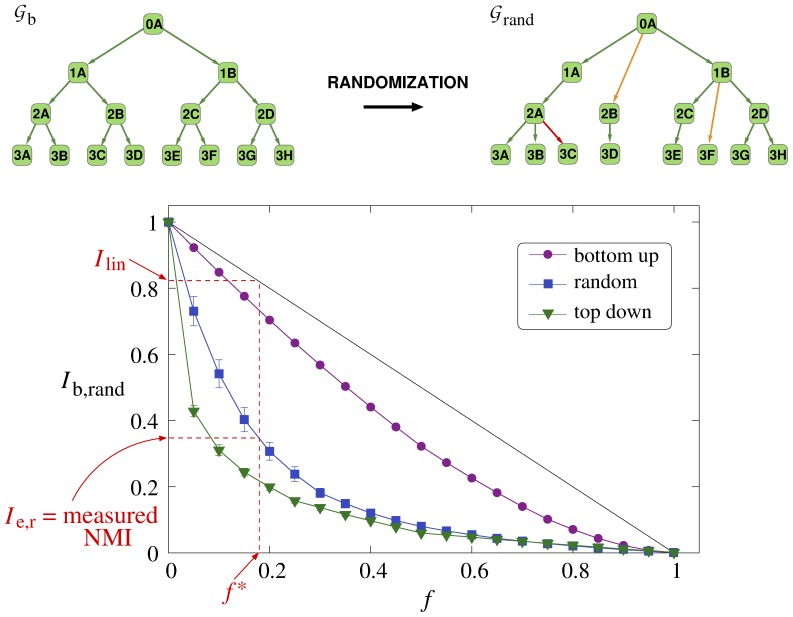
Using the normalized mutual information (NMI) for measuring the similarity between hierarchies. We tested the behavior of the NMI by applying (1) to a binary tree of 1,023 nodes, 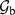, and its randomized counter part, 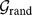, obtained by rewiring the links at random, as shown in the illustration at the top. The decay of the obtained NMI is shown in the bottom panel as a function of the fraction of the rewired links, 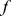. The three different curves correspond to rewiring the links in reverse order according to their position in the hierarchy (purple circles), rewiring in random order (blue squares) and rewiring in the order of the position in the hierarchy (green triangles). The concept of the linearized mutual information (LMI) for the general tag hierarchy reconstruction problem is illustrated in red: By projecting the measured 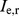 value onto the 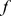 axis via the blue curve we obtain 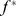, giving the fraction of rewired links in a randomization process with the same NMI value. The LMI is equal to 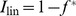, corresponding to the fraction of unchanged links.

We examined the behavior of the NMI given in (1) by taking a binary tree of 1,023 nodes, 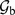, and comparing it to its randomized counterpart, 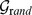, obtained by rewiring a fraction of 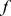 links to a random location. In Fig.1. we show the measured NMI as a function of 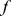. If we start the rewiring with links pointing to leafs, and continue according to the reverse order in the hierarchy, the NMI shows a close to linear decay as a function of 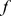 almost in the entire 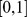 interval (purple circles). However, if links are chosen in random order, 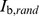 is decreasing much faster in the small 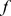 region, with an overall non-linear 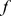 dependency (blue squares). An even steeper decay can be observed when links are chosen in the order of their position in the hierarchy (green triangles). Nevertheless, 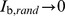 when 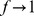 in all cases, thus, the similarity defined in this way is vanishing for a pair of independent DAGs. Meanwhile, the significant difference between the three curves displayed in Fig.1c shows that the NMI is sensitive also to the position of the rewired links in the hierarchy: rewiring the top levels of the hierarchy is accompanied by a drastic drop in the similarity, while changes at the bottom of the hierarchy cause only a minor decrease, which is linear in the fraction of rewired links.

This non-trivial feature of the NMI allows the introduction of another interesting quality measure for a reconstructed hierarchy. Supposing a similar randomization procedure on 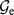 as shown in Fig.1, we may ask what fraction of links has to be rewired on average for reaching the same NMI as 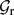
? The formal definition of this measure is given as follows. Let 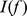 denote the average NMI obtained for a fraction of 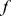 randomly rewired links, where the links are chosen in random order, 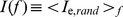. By projecting the NMI between the exact- and reconstructed hierarchies, 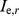, to the 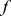 axis using this function as 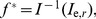
(2) we receive the fraction of randomly chosen links to be rewired in 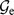 for obtaining a randomized hierarchy with the same NMI as 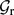, (see Fig.1 for illustration). Based on that we define the linearized mutual information, (LMI) as 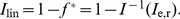
(3)


This quality measure corresponds to the fraction of unchanged links in a random link rewiring process, resulting in a hierarchy with the same NMI as 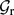. (The reason for calling it “linearized” is that (3) is actually projecting 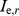 to the linear 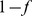 curve). By comparing the LMI to the fraction of exactly matching links, 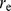, we gain further information on the nature of the reconstructed DAG: If 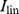 is significantly larger than 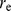, the reconstructed DAG is presumably better for the links high in the hierarchy, whereas if 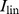 is significantly lower than 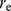, the reconstructed DAG is more precise for links close to the leafs.

### Real tagging systems

#### Reconstructing the hierarchy of protein functions

Although the primary targets of tag hierarchy extraction methods are given by tagging systems with no pre-defined hierarchy between the tags, for testing the quality of the extracted hierarchy we need input data for which the exact hierarchy is also given. A very important real tag hierarchy is provided by protein functions as described in the Gene Ontology [56], organizing function annotations into three separate DAGs corresponding to “biological process”, “molecular function” and “cellular component” oriented description of proteins. The corresponding input data for a tag hierarchy extraction algorithm would be a collection of proteins, each tagged by its function annotations. Luckily, the Gene Ontology provides also a regularly updated large data set enlisting proteins and their known functions aggregated from a wide range of sources, (a more detailed description of the data set we used is given in Materials and Methods).

**Figure 2:**
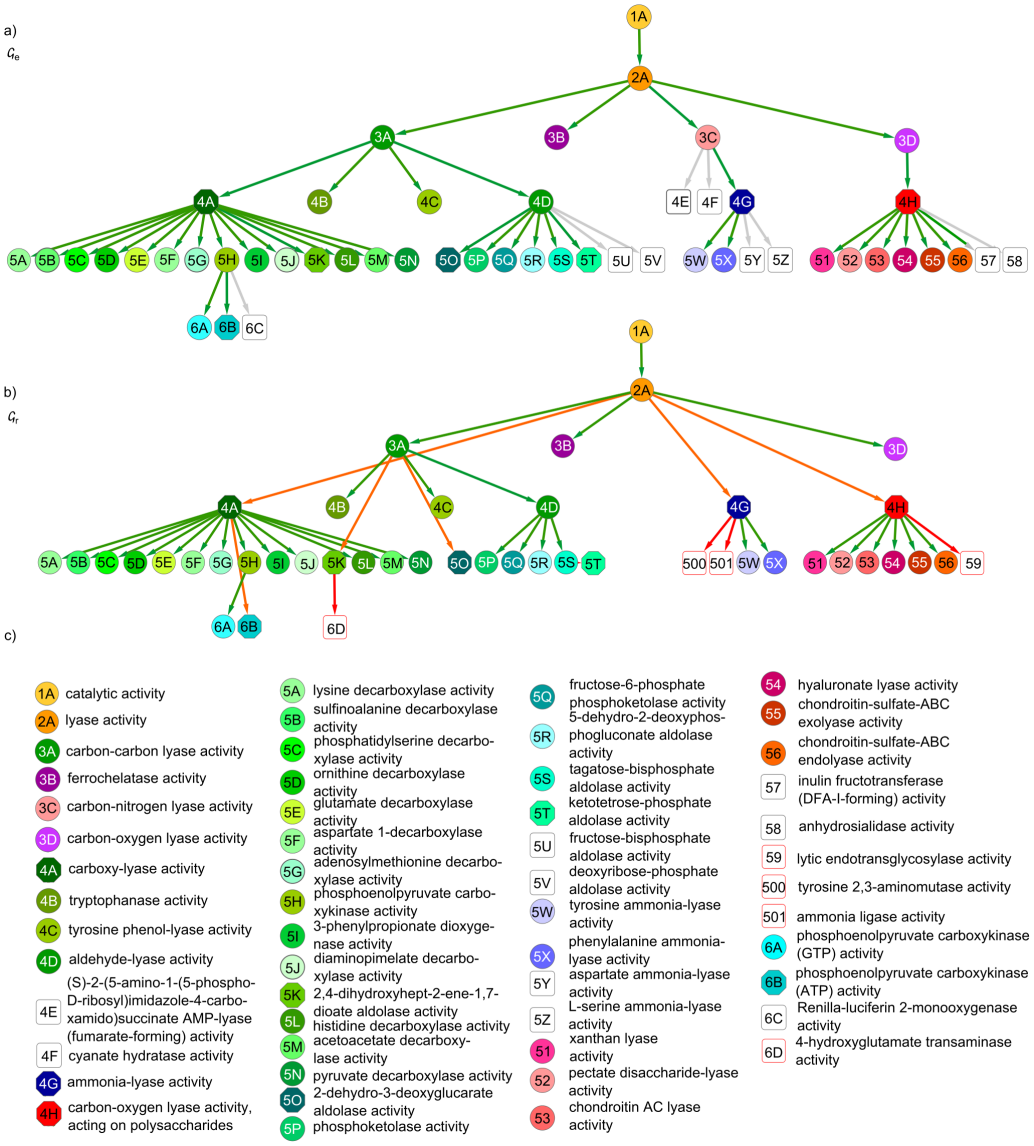
Comparison between the exact hierarchy and the reconstructed hierarchy obtained from algorithm A. a) A subgraph in the hierarchy of protein functions, (describing molecular functions), according to the Gene Ontology, treated as the exact hierarchy, 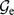. b) The hierarchy between the same tags obtained from running algorithm A on the tagged protein data set, (the reconstructed hierarchy, 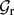). The exactly matching- and acceptable links are colored green and orange respectively, the unrelated links are shown in red, while the missing links are colored gray. c) The list of included protein functions in panels (a) and (b).

In Fig.2a we show a smaller subgraph from the hierarchy between molecular functions given in the Gene Ontology, 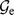, together with the subgraph between the same tags in the result obtained by running our algorithm A on the tagged protein data set, 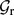, displayed in Fig.2b. The matching between the two subgraphs is very good: the majority of the connections are either exactly the same (shown in green), or acceptable (shown in orange), by-passing levels in the hierarchy and e.g., connecting “grandchildren” to “grandparents”. The appearing few unrelated– and missing links are colored red and gray, respectively.

The quality measures obtained for the complete reconstructed hierarchy are given in table 1. For comparison we also evaluated the same measures for algorithm B, the algorithm by P. Heymann and H. Garcia-Molina, and the algorithm by P. Schmitz. According to the results all 4 methods perform rather well, however, our algorithm seems to achieve the best scores. Although the ratio of exactly matching links is 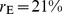, (which is not very high), the ratio of acceptable links is reaching 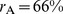, which is very promising. The NMI given by (1) is 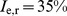, however, the LMI according to (3) is 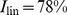. (The corresponding plot showing the decay of the NMI between the Gene Ontology hierarchy and its randomized counterpart is given in Sect.S2.2 in File S1). Thus, the similarity between our reconstructed hierarchy and the hierarcy from the Gene Ontology is so high that if we would randomize the Gene Ontology, (by rewirnig the links in random order), the same NMI value would be reached already after rewiring 22% of the links. The large difference between 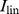 and 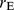 in favour of 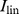 indicates that our algorithm is better at predicting links higher in the hierarchy. E.g., in a randomization with random link rewiring order keeping only 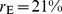 of the links unchanged, the NMI would be around 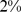 instead of the actualy measured 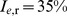. The reason why 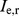 can stay relatively high for the reconstructed hierarchy is that the majority of the non-matching links are low in the hierarchy, therefore, have a smaller effect on the NMI.

**Table 1:** Quality measures for the reconstructed hierarchies in case of the protein function data set.

	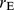	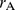	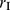	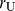	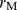	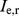	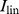
algorithm A	21%	66%	2%	32%	0%	35%	78%
algorithm B	20%	52%	3%	44%	1%	30%	75%
P. Heymann & H. Garcia-Molina	19%	51%	3%	46%	0%	30%	75%
P. Schmitz	18%	65%	2%	23%	10%	30%	75%

The quality of the tag hierarchy obtained for the tagged protein data set, 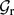, was evaluated by comparing it to the hierarchy of protein functions in the Gene Ontology, 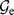. The quality measures presented in the different columns are the following: the ratio of exactly matching links in 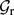,denoted by 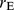, the ratio of acceptable links, 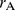, (connecting more distant ancestor-descendant pairs), the ratio of inverted links, 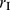, (pointing in the opposite direction), the ratio of unrelated links, 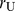, (connecting tags on different branches in 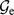), the ratio of missing links in 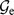, denoted by 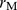, the normalized mutual information between the two hierarchies, 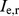, and the linearized mutual information, 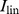, corresponding to the fraction of exactly matching links remaining after a random link rewiring process stopped at NMI value given by 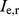. The different rows correspond to results obtained from algorithm A (1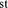 row), algorithm B (2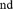 row),the method by P. Heymann & H. Garcia-Molina (3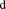 row), and the algorithm by P. Schmitz (4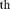 row).

#### Hierarchy of Flickr tags

One of the most widely known tagging systems is given by Flickr, an online photo management and sharing application, where users can tag the uploaded photos with free words. Since the tags are not organized into a global hierarchy, this system provides an essential example for the application field of tag hierarchy extracting algorithms. We have run our algorithm B on a relatively large, filtered sample of photos, (the details of the construction of our data set are given in Methods). Although the “exact” hierarchy between the tags is not known in this case, since the tags correspond to English words, we can still give a qualitative evaluation of the result just by looking at smaller subgraphs in the extracted hierarchy.

**Figure 3:**
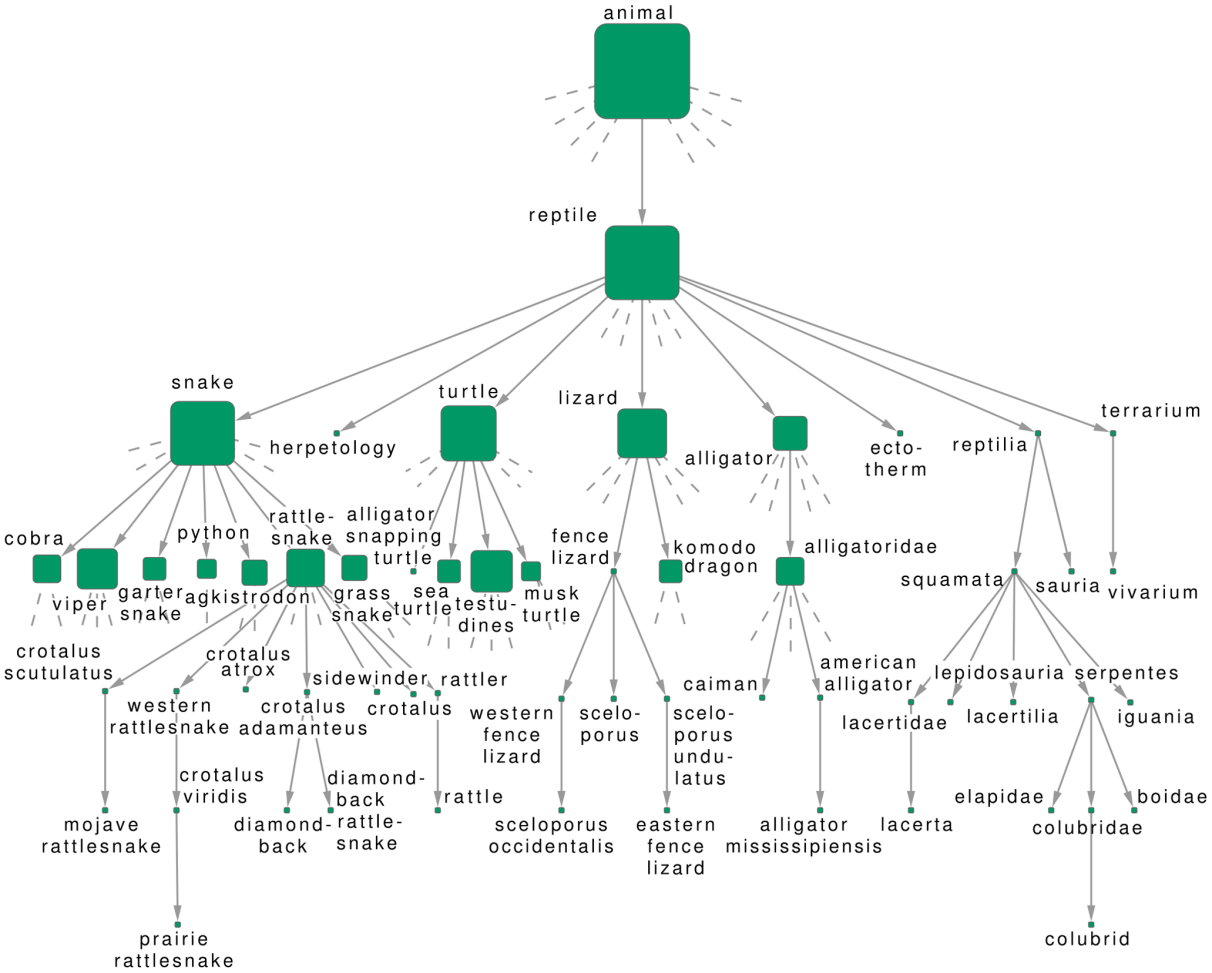
Subgraph from the hierarchy between Flickr tags. By running our algorithm B on a filtered sample from Flickr, we obtained a hierarchy between the tags appearing on the photos in the sample. Since the total number of tags in our data reached 25,441, here we show only a smaller subgraph from the result, corresponding to a part of the tags categorized under “reptile”. Stubs correspond to further direct descendants not shown in the figure, and the size of the nodes indicate the total number of descendants on a logarithmic scale, (e.g., “prairie rattlesnake” has none, while “snake” has altogether 110.).

An example is given in Fig.3., showing a few descendants of the tag “reptile” in our reconstruction. Most important direct descendants are “snake”, “lizard”, “alligator” and “turtle”. The tags under these main categories seem to be correctly classified, e.g., “alligator snapping turtle” is under “turtle”, (instead of the also related “alligator”). Interestingly, Latin names (binomial names) from the taxonomy of “reptilia” form a further individual branch under “reptile”, however, occasionally we can also see binomial names directly connected to the corresponding English name of the given species. More examples from our result on the Flickr data are given in Sect.S3.1 in File S1, which taken together with Fig.3 give an overall impression of a meaningful hierarchy, following the “common sense” by and large. (Furthermore, similar samples from the hierarchies extracted by the other methods are also given in Sect.S3.2 in File S1.)

#### Hierarchy of IMDb tags

**Figure 4:**
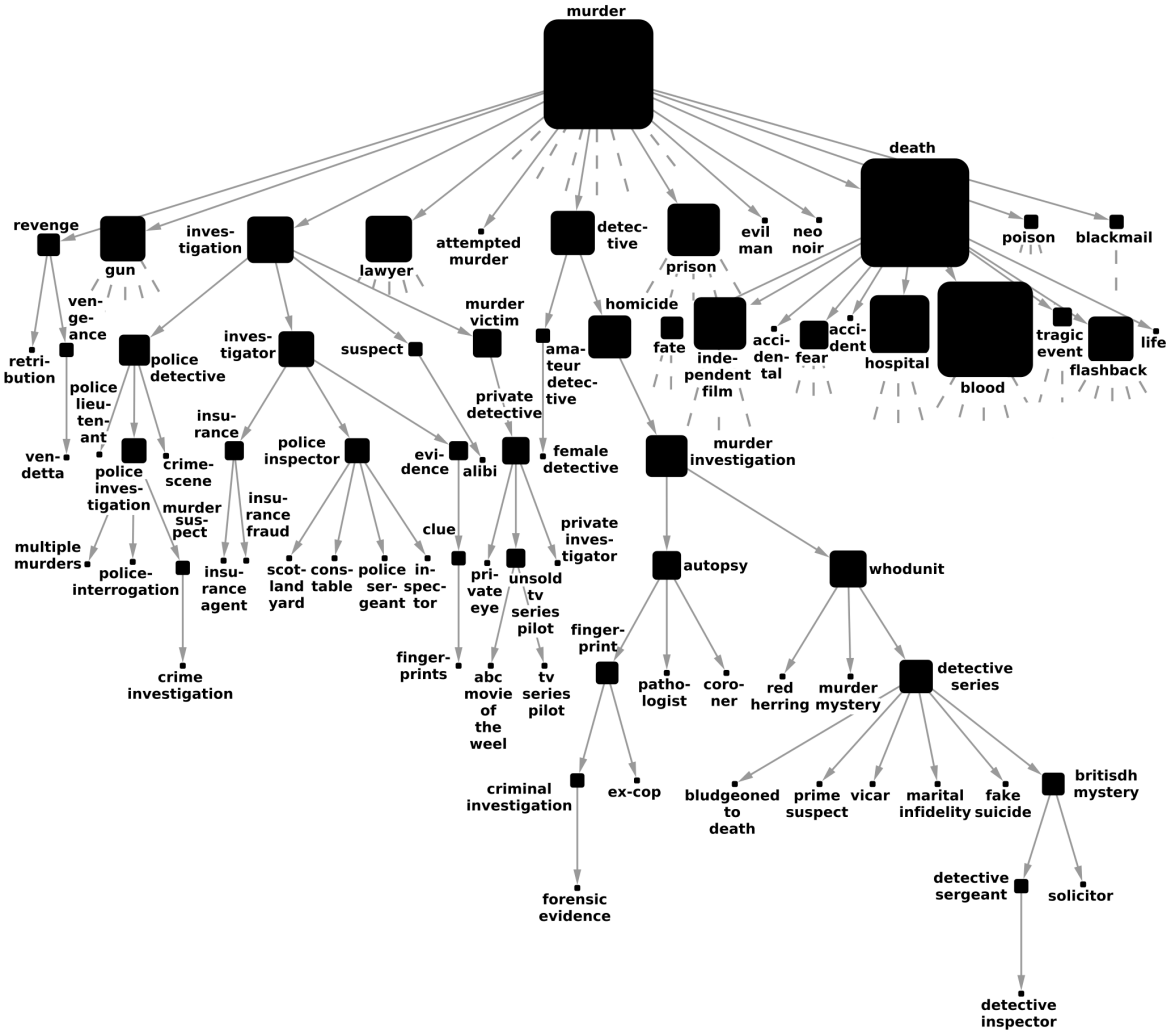
Subgraph from the hierarchy between IMDb tags. The results were obtained by running Algorithm B on a filtered sample of films from IMDb, tagged by keywords describing the content of the movies. Here we show only a smaller subgraph between the descendants of “murder”, where stubs correspond to further direct descendants not shown in the figure, and the size of the nodes indicate the total number of descendants on a logarithmic scale.

Another widely known online database is given by the IMDb, providing detailed information related to films, television programs and video games. One of the features relevant from the point of view of our research is that keywords related to the genre, content, subject, scenes, and basically any relevant feature of the movies are also available. These can be treated similarly to the Flickr tags, i.e., they are corresponding to English words, which are not organized into a hierarchy. In Fig.4. we show results obtained by running Algorithm B on a relatively large, filtered sample of tagged movies. (The details of the construction of the data set are given in Methods). Similarly to the Flickr data, we display a smaller part of the branch under the tag “murder” in the extracted hierarchy. Most important direct descendants are corresponding to “death”, “prison” and “investigation”, with “blood”, “suspect” and “police detective” appearing on lower levels of the hierarchy. Although the tags appearing in the different sub-branches are all related to their parents, the quality of the Flickr hierarchy seemed a bit better. This may be due to the fact that keywords can pertain to any part of the movies, and hence, the tags on a single movie can already be very diverse, providing a more difficult input data set for tag hierarchy extraction. Nevertheless, this result reassures our statement related to the Flickr data, namely that the hierarchies obtained from our algorithm have a meaningful overall impression. (Similar samples from the hierarchies obtained with the other methods are shown in Sect.S3.2 in File S1.)

### Synthetic benchmark based on random walks

#### Defining the benchmark system

Providing adjustable benchmarks is very important when testing and comparing algorithms. The basic idea of a benchmark in general is given by a system, where the ground truth about the object of search is also known. However, for most real systems this sort of information is not available, therefore, synthetic benchmarks are constructed. E.g., community finding is one of the very intensively studied area of complex network research, with an enormous number of different community finding algorithms available [25]. Since the ground truth communities are known only for a couple of small networks, the testing is usually carried out on the LFR benchmark [27], which is a purely synthetic, computer generated benchmark: the communities are pre-defined, and the links building up the network are generated at random, with linking probabilities taking into account the community structure. The drawback of such synthetic test data is its artificial nature, however, the benefit on the other side is the freedom of the choice of the parameters, enabling the variance of the test conditions on a much larger scale compared to real systems.

Here we propose a similar synthetic benchmark system for testing tag hierarchy extraction algorithms. The basic idea is to start from a given pre-defined hierarchy, (the “exact” hierarchy), and generate collections of tags at random, (corresponding to tagged objects in a real system), based on this hierarchy. The tag hierarchy extraction methods to be tested can be run on these sets of tags, and the obtained hierarchies, (the "reconstructed" hierarchies), can be compared to the exact hierarchy used when generating the synthetic data. When drawing an analogy between this system and the LFR benchmark, our pre-defined hierarchy is corresponding to the pre-defined community structure in the LFR benchmark, while the generated collections of tags are corresponding to the random networks generated according to the communities.

**Figure 5:**
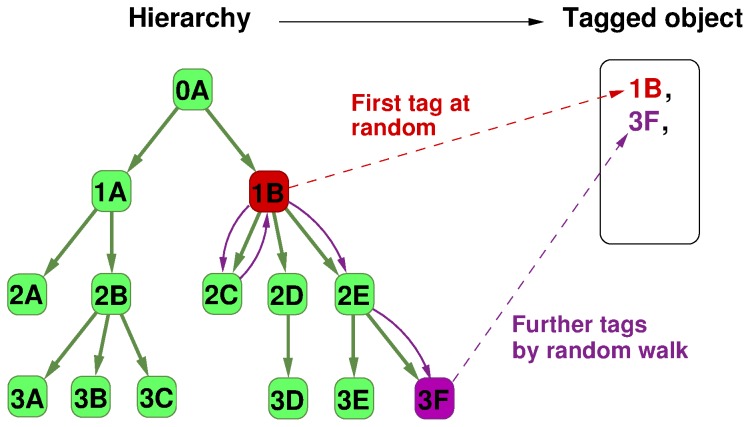
Generating tags on virtual objects by random walks on the hierarchy. The objects in this approach are represented simply by collections of tags. For a given collection, the first tag is picked at random, (illustrated in red), while the rest of the tags are obtained by implementing a short undirected random walk on the DAG, starting from the first tag, (illustrated in purple).

To make the above idea of a synthetic tagging system work in practice, we have to specify the method for generating the random collections of tags based on the given pre-defined hierarchy. In general, the basic idea is that tags more closely related to each other according to the hierarchy should appear together with a larger probability compared to unrelated tags. To implement this, we have chosen a random walk approach as suggested in [58]. The first tag in each collection is chosen at random. For the rest of the tags in the same collection, with probability 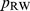 we start a short undirected random walk on the hierarchy starting from the first tag, and choose the endpoint of the random walk, or with probability 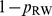 we again choose at random. An illustration of this process is given in Fig.5, (a brief pseudo-code of the data generation algorithm is given in Algorithm S4 in File S1). The parameters of the benchmark are the following: the pre-defined hierarchy between the tags, the frequency of the tags when choosing at random, the probability 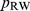 for generating the second and further tags by random walk, the length of the random walks, the number of objects and finally, the distribution of the number of tags per object. Although this is a long list of parameters, the quality of the reconstructed hierarchy is not equally sensitive to all of them. E.g., according to our experiments change in the topology of the exact hierarchy, or in the length of the random walk have only a minor effect, while the distribution of the tag frequencies seems to play a very important role.

### Results on synthetic data

In Table 2. we show the tag hierarchy extraction results obtained on synthetic data generated by using our random walk based benchmark system. In the data generation process the exact hierarchy was set to a binary tree of 1,023 tags, with tag frequencies decreasing linearly as a function of the depth in the hierarchy. We generated an average number of 3 co-occurring tags on altogether 2,000,000 hypothetical objects, with random walk probability of 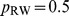 and random walk lengths chosen from a uniform distribution between 1 and 3. We ran the same algorithms on the obtained data as in case of the protein data set, and used the same measures for evaluating the quality of the results. According to Table 2., the majority of the algorithms perform very well, e.g., algorithm B and the algorithm by P. Heymann & H. Garcia-Molina are producing almost perfect reconstructions, thus, this example is an “easy” data set. Interestingly, the results of the algorithm by Schmitz were very poor on this input. Nevertheless, this method is still competitive with the others, e.g., it showed a quite good performance on the protein data set. However, the study of why does this algorithm behave completely different from the others on our benchmark is out of the scope of the present work.

**Table 2:** Quality measures of the reconstructed hierarchies for the “easy” synthetic data set.

	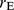	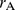	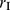	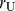	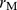	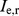	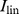
algorithm A	67%	00%	0%	0%	0%	91%	99%
algorithm B	100%	100%	0%	0%	0%	100%	100%
P. Heymann & H. Garcia-Molina	99%	100%	0%	0%	0%	93%	99%
P. Schmitz	0%	0%	0%	0%	100%	0%	0%

When generating the data set, the frequency of the initial tags was decreasing linearly as a function of the level depth in the exact hierarchy. We show the same quality measures as in Table 1.: the ratio of exactly matching links, 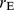, the ratio of acceptable links, 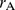, the ratio of inverted links, 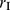, the ratio of unrelated links, 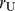, the ratio of missing links, 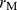, the normalized mutual information between the exact- and the reconstructed hierarchies, 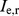, and the linearized mutual information, 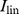. The different rows correspond to results obtained from algorithm A, (1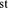 row), algorithm B, (2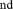 row), the method by P. Heymann & H. Garcia-Molina (3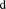 row), and the algorithm by P. Schmitz (4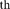 row).

The “easy” synthetic data discussed above can be turned into a “hard” one by changing the frequency distribution of the tags. In Table 3. we show the results obtained when the tag frequencies were independent of the level depth in the hierarchy, and had a power-law distribution, with the other parameters of the benchmark left unchanged. According to the studied quality measures, the performance of the involved methods drops down drastically compared to Table 2. However, algorithm B provides an exception in this case, achieving pretty good results even for this “hard” test data. E.g., the NMI value is still 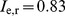 for our algorithm, while for e.g., the algorithm by P. Heymann & H. Garcia-Molina it is reduced to 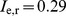. Moreover, the fraction of exactly matching links is almost 90% for algorithm B, while it is below 50% for the algorithm by P. Heymann & H. Garcia-Molina. This shows that algorithm B can have a significantly better performance compared to other algorithms, as the quality of its output is less dependent on the correlation between tag frequencies and level depth in the hierarchy. Another interesting effect in Table 2. is that the results for the algorithm by Schmitz are slightly better compared to the “easy” data set. As we mentioned earlier, studies of the reasons for the outlying behavior of this algorithm on our benchmark compared to the other methods is left for future work.

**Table 3:** Quality measures of the reconstructed hierarchies for the “hard” synthetic data set.

	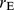	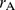	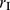	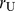	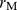	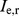	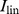
algorithm A	31%	5%	18%	47%	0%	18%	66%
algorithm B	89%	91%	6%	3%	0%	83%	97%
P. Heymann & H. Garcia-Molina	48%	54%	29%	17%	0%	29%	76%
P. Schmitz	1%	2%	1%	3%	94%	1%	5%

In this case the frequency of the initial tags was independent of their position in the exact hierarchy during the benchmark generation, and the frequency distribution followed a power-law. This change compared to the data set used in Table 2. results in significant decrease in the quality measures for most of the involved methods, as shown by the ratio of acceptable links, 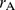, the ratio of inverted links, 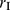, the ratio of unrelated links, 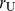, the ratio of missing links, 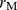, the normalized mutual information between the exact- and the reconstructed hierarchies, 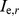, and the linearized mutual information, 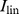. The different rows correspond to results obtained from algorithm A, (1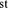 row), algorithm B, (2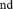 row), the method by P. Heymann & H. Garcia-Molina (3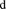 row), and the algorithm by P. Schmitz (4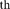 row).

The effects of the modifications in the other parameters of the benchmark are discussed in Sect.S4.2-S4.3 in File S1. Nevertheless these results already show that the provided framework can serve as versatile test tool for tag hierarchy extraction methods.

## Methods

### 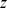-score

Both algorithms introduced in the paper depend on the 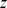-score related to the number of co-occurrences between a pair of tags. If the tags are assigned to the objects completely at random, the distribution of the number of co-occurrences for a given pair of tags 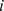 and 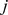 follows the hypergeometric distribution: Assuming that tag 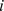 and 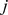 appear altogether on 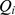 and 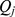 objects respectively, let us consider the random assignment of tag 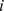 among a total number of 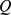 objects. This is equivalent to drawing 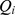 times from the objects without replacement, where the “successful” draws correspond to objects also having tag 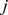, (and the total number of such objects is 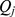). Based on this, the probability for observing a given 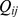 number of co-occurrences between 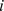 and 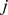 is 
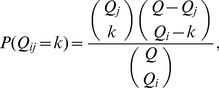
(4) with the expected number of co-appearances given by 
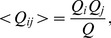
(5) and the variance formulated as 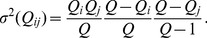
(6)


The 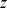-score is defined as the difference between the observed number of co-occurrences in the data, 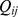, and the expected number of co-occurrences at random as given in (5), scaled by the standard deviation according to (6), 
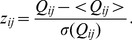
(7)


### Normalized mutual information

For discrete variables 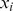 and 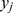 with a joint probability distribution given by 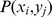, the mutual information is defined as 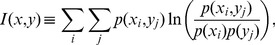
(8) where 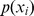 and 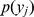 denote the (marginal) probability distributions of 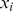 and 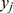 respectively. If the two variables are independent, 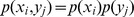, thus, 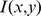 becomes 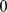. The above quantity is very closely related to the entropy of the random variables, 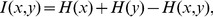
(9) where 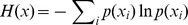 and 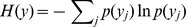 correspond to the entropy of 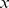 and 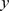, while 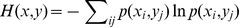 denotes the joint entropy. Based on (9), the NMI can be defined as 
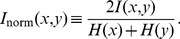
(10)


This way the NMI is 1 if and only 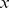 and 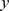 are identical, and 0 if they are independent.

### Data

#### Protein data

Both the exact DAG describing the hierarchy between protein functions and the corresponding input data set given by proteins tagged with known function annotations were taken from the Gene Ontology [56]. The hierarchy of protein function is composed of three separate DAGs, corresponding to “molecular function”, “biological process” and “cellular component”. We concentrated on molecular functions, where the complete DAG has altogether 6,469 tags. However, a considerable part of these annotations are rather rare, thus, reconstructing the complete hierarchy would be a very hard task due to the lack of information. Therefore, we took a smaller subgraph, namely the branch starting from “catalytic activity”, counting 4,181 tags, most of which are relatively more frequent.

For the data set of proteins, tagged with their known molecular function annotations, we took the monthly (quality controlled) release as in 2012.08.01. For simplicity, we neglected proteins lacking any tags appearing in the exact hierarchy, and deleted all annotations which are not descendants of “catalytic activity”. The resulting smaller data set contained 5,913,610 proteins, each having on average 3.7 tags. This data set, (together with the corresponding exact DAG) is available at (http://hiertags.elte.hu).

### Flickr data

Flickr provides the possibility for searching photos by tags, thus, as a first step we downloaded photos resulting from search queries over a list of 68,812 English nouns, yielding altogether 2,565,501 photos, (the same photo can appear multiple times as a result for the different queries). At this stage we stored all the tags of the photos and the anonymous user id of the photo owners as well. Next, the set of tags on the photos had to be cleaned: only English nouns were accepted, and in case of parts of a compound word appeared beside the compound word on the same photo, the smaller parts were deleted, leaving only the complete compound word. Since our algorithms rely on the weighted network of co-appearances, we applied a further filtering: a link was accepted only if the corresponding tags co-appeared on photos belonging to at least 10 different users. The resulting tag co-appearance network had 25,441 nodes, encoding information originating from 1,519,030 photos. We made the list of weighted links between the tags available at (http://hiertags.elte.hu).

### IMDb data

We have downloaded the data from the IMDb Web site[59], and used the “keywords.list.gz” data file, listing the keywords associated with the different movies. The goal of the keywords is helping the users in searching amongst the movies, and keywords can pertain to any part, scene, subject, gender, etc. of the movie. Although keywords can be given only by registered users, there is no restriction what so ever for registering, and the submitted information is processed by the "Database Content Team" of the IMDb site. The version of the original data we are used here contained 487,356 movie titles and 136,204 different keywords. However, to improve the quality of the data set, we restricted our studies to keywords appearing on at least a 100 different movies, leaving 336,223 movies and 6,358 different keywords in the data set. This cleaned version is available at (http://hiertags.elte.hu).

## Discussion

We introduced a detailed framework for tag hierarchy extraction in tagging systems. First, we have defined quality measures for hierarchy extraction methods based on comparing the obtained results to a pre-defined exact hierarchy. A part of these quantities were simply given by fractions of links fulfilling some criteria, (e.g., exactly matching, inverted, etc.). However we also defined the NMI between the exact- and the reconstructed hierarchies, providing a quality measure which is sensitive also to the position of the non-matching links in the hierarchy. This was illustrated by our experiments comparing a hierarchy to its randomized counterpart, where the NMI showed a significantly faster decay when the rewiring was started at the top of the hierarchy, compared to the opposite case of starting from the leafs.

Furthermore, we developed a synthetic, computer generated benchmark system for tag hierarchy extraction. This tool provides versatile possibilities for testing hierarchy extraction algorithms under controlled conditions. The basic idea of our benchmark is generating collections of tags associated to virtual objects based on a pre-defined hierarchy between the tags. By running a tag hierarchy extraction algorithm on the generated synthetic data, the obtained result can be compared to the pre-defined exact hierarchy used in the data generation process. According to our experiments on the benchmark, by changing the parameters during the generation of the synthetic data, we can enhance or decrease the difficulty of the tag hierarchy reconstruction.

In addition, we developed two novel tag hierarchy extraction algorithms based on the network approach, and tested them both on real systems and computer generated benchmarks. In case of the tagged protein data the similarity between the obtained protein function hierarchy and the hierarchy given by the Gene Ontology was very encouraging, and the hierarchy between the English words obtained for the Flickr and IMDb data sets seemed also quite meaningful. The computer generated benchmark system we have set up provides further possibilities for testing tag hierarchy extraction algorithms in general. By changing the parameters during the input generation we can enhance or decrease the difficulty of the tag hierarchy reconstruction.

Our methods were compared to current state of the art tag hierarchy extraction algorithms by P. Heymann & H. Garcia-Molina and by P. Schmitz. Interestingly, the rank of the algorithms according to the introduced quality measures was varying from system to system. In case of the protein data set algorithm A was slightly ahead of the others, while the rest of the methods achieved more or less the same quality. In turn, for the easy synthetic test data, algorithm B and the algorithm by P. Heymann & H. Garcia-Molina reached almost perfect reconstruction, with algorithm A left slightly behind, and the algorithm by P. Schmitz achieving very poor marks. However, when changing to the hard synthetic test data, a large difference was observed between the quality of the obtained results, as algorithm B significantly outperformed all other methods.

The different ranking of the algorithms for the included examples indicates that tag hierarchy extraction is a non-trivial problem where a system can be challenging for one given approach and easy for another method and vice versa. Nevertheless the results obtained indicate that tag hierarchy extraction is a very promising direction for further research with a great potential for practical applications.

## Supporting Information

File S1 **File S1 provides more details on our algorithms and similarity measures, together with further results on the studied real systems and the synthetic benchmark.**
(PDF)
