# Supplementary material for: Extracting Tag Hierarchies
Source: PLoS One. 2013 Dec 31;8(12):e84133. doi: 10.1371/journal.pone.0084133 (PMC3877228; doi:10.1371/journal.pone.0084133)
Supplement: File S1 — provides more details on our algorithms and similarity measures, together with further results on the studied real systems and the synthetic benchmark. (PDF) [file pone.0084133.s001.pdf]

Extracting tag-hierarchies
Supporting Information

S1 Algorithms

S1.1 Algorithm A

S1.1.1 Complexity

The pseudo code of the algorithm would be to long to be displayed in a single page, thus, we divided it into two parts. The first part, corresponding to the preparation of the weighted network between the tags and the building of local hierarchies is given in Algorithm S1. By assuming that the number of tags on

Algorithm S1 Algorithm A, 1st part: building local hierarchies.

```
for all objects: object1 do
    for all tags appearing on object1: tag1 do
        for all tags appearing on object1: tag2 do coappearances(tag1,tag2)+=1
        end for
    end for
end for
for all tags: tag1 do
    max= maximal coappearances(tag1,tag2)
    for all tags: tag2 do
        if coappearances(tag1, tag2) >= ω * max then
            calc zscore(tag1,tag2)
            strongpartners(tag1, tag2) = zscore(tag1, tag2)
        end if
    end for
end for
for all tags: tag1 do
    parent = undef
    for all strongpartners of tag1 sorted to descending order: tag2 do
        if parent = undef and not exists strongpartners(tag2, tag1) then
            parent = tag2
        end if
    end for
end for
```

one object is $\mathcal{O}(1)$, the number of operations needed for generating the weighted co-occurrence network between the tags can be given by the number of objects, Q, as $\mathcal{O}(Q)$. According to our experience, the resulting co-occurrence network between the tags is usually sparse, thus, the number of links in the network between the tags, M, and the number of tags, N, are similar in magnitude, $\mathcal{O}(M) = \mathcal{O}(N)$, and the average number of links of the tags is $\mathcal{O}(1)$. According to that, the individual thresholding of the network based on the strongest link on each tag also needs $\mathcal{O}(N \log N)$ operations. Similarly, the calculation of the z-score and choosing the in-neighbor with the highest value as a parent need also $\mathcal{O}(M \log M)$ operations.

In the next phase, the smaller isolated subgraphs under the local roots have to be assembled into a single hierarchy, as shown in Algorithm S2. Choosing the global root of the hierarchy needs $\mathcal{O}(N)$ operations,

Algorithm S2 Algorithm A, 2nd part: assembly into a global hierarchy.

```
if there are more components then
    for all roots: root do
        h(root) = entropy of root
        for all tags in the component of root: tag1 do
            component(tag1) = root
        end for
    end for
    global_root = root with highest entropy
    for all roots except the global: root do
        suggested_parent(root) = undef
        for all coappearing tags sorted to descending coappearances: tag2 do
            if suggested_parent(root) = undef and component(tag2) is not root then
                suggested_parent(root) = tag2
            end if
        end for
    end for
    for all roots appearing in suggested_parent: root do
        tag1 = root
        empty visited
        while does not exists visited(tag1) and exists suggested_parent(tag1) do
            tag1 = component(suggested_parent(tag1))
            visited(tag1) = 1
        end while
        if exists visited(tag1) then
            for all roots in visited: root2 do looped(root2) = 1 delete suggested_parent(root2)
            end for
        end if
    end for
    for all roots in looped, sorted to descending order of h: root do
        for all tags coappearing with root: tag1 do
            if not exists suggested_parent(root) then
                check whether tag1 is below root
                if tag1 is not below root then suggested_parent(root) = tag1
                end if
            end if
        end for
        if not exists suggested_parent(root) then suggested_parent(root) = global_root
        end if
    end for
end if
```

and similarly, choosing the parent of a local root also needs at most $\mathcal{O}(N)$ operations. During this process we need to detect (and correct) possible newly created loops, requiring at most $\mathcal{O}(N)$ operations. Based on the above, the resulting overall complexity of algorithm A is $\mathcal{O}(Q)+\mathcal{O}(M\log M) = \mathcal{O}(Q)+\mathcal{O}(N\log N)$, where we assumed that the co-occurrence network between the tags is sparse, i.e., $\mathcal{O}(M) = \mathcal{O}(N)$.

S1.1.2 Optimizing the parameter ω

The parameter $\omega \in [0, 1]$ in algorithm A is corresponding to the local weight threshold used for throwing away weak connections in the tag co-occurrence network. In order to find the optimal value for ω, we measured the LMI between the reconstructed hierarchy and the exact hierarchy as a function of ω in case of the protein function data set. The results of this experiment are shown in Fig.S1. Although $I_{\rm lin}$ is showing only minor changes over the whole range of possible ω values, a maximal plateau can still be observed between $\omega = 0.3$ and $\omega = 0.55$. Based on this, throughout the experiments detailed in our paper, we used algorithm A with $\omega = 0.4$.

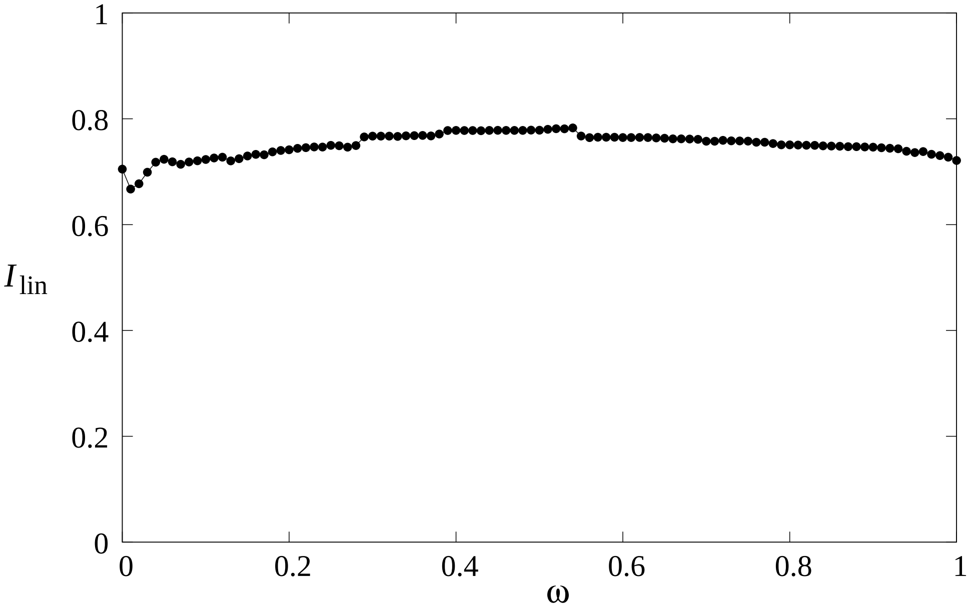

Figure S1. Optimizing the parameter ω. We show the LMI as a function of ω for the protein function data set.

S1.2 Algorithm B

S1.2.1 Complexity

The pseudo code for the algorithm is given in Algorithm S3. The preparation of the tag co-occurrence network is the same as in case of algorithm A, with a complexity of $\mathcal{O}(Q)$, and similarly, the calculation of the z-score needs $\mathcal{O}(M)$ operations. To evaluate the eigenvector centrality, we simply use the power iteration method on the filtered co-appearance matrix, (see the pseudo code), which needs $\mathcal{O}(N)$ operations, for the typical case of a sparse matrix. The hierarchy is assembled bottom up, and the calculation of the scores for the possible parents of a given tag requires $\mathcal{O}(N \cdot \log N)$ operations, assuming that the structure of the complete DAG is similar to a tree with a constant branching number. (In case it is chain-like, this is modified to $\mathcal{O}(N^2)$, whereas for a star-like topology, it is only $\mathcal{O}(N)$). The resulting overall complexity of the algorithm is $\mathcal{O}(Q) + \mathcal{O}(N \cdot \log N)$.

Algorithm S3 Algorithm B

```
for all tags: tag1 do
    for all tags: tag2 do
        calc zscore(tag1, tag2)
    end for
end for
for all tags: tag1 do
    for all tags: tag2 do
        if zscore(tag1, tag2) > threshold_B or coappearances(tag1, tag2) >= 0.5 * objects(tag1) or coappearances(tag1, tag2) >= 0.5 * objects(tag2) then
            M(tag1, tag2) = coappearances(tag1, tag2)
            strength(tag1) += coappearances(tag1, tag2)
        end if
    end for
end for
for all tags: tag1 do
    centrality(tag1) = strength(tag1)
end for
for i=1, i<=100 do
    sum = 0
    for all tags: tag1 do
        for all tags: tag2 do
            temp_centrality(tag1) = M(tag1, tag2) * centrality(tag2)
        end for
        sum += temp_centrality(tag1)
    end for
    for all tags: tag1 do centrality(tag1) = temp_centrality(tag1) / sum
    end for
end for
for tags sorted to ascending centralities: tag1 do
    empty score;
    for coappearing partners of tag1: tag2 do
        score(tag2) = zscore(tag1, tag2)
    end for
    for descendants of tag1: desc do
        for coappearing partners of desc: tag2 do
            if tag2 coappears with tag1 and centrality(tag2) ¿ centrality(tag1) and (zscore(tag1, tag2) > threshold_B or coappearances(tag1, tag2) >= 0.5 * objects(tag1)) and (zscore(desc, tag2) > threshold_B or coappearances(desc, tag2) >= 0.5 * objects(desc)) then
                score(tag2) += zscore(desc, tag2)
            end if
        end for
    end for
    if score is not empty then
        parent(tag1) = highest scoring tag
    end if
end for
```

S1.2.2 Optimizing the z-score threshold

The z-score threshold is an important parameter in algorithm B, which is used for pruning the network of co-occurrences between the tags by throwing away irrelevant connections. In order to optimize this parameter, we have run tests on the "hard" synthetic data set, introduced in Section "Results on synthetic data" in the main paper. The reason for this choice instead of, e.g., the protein function data set as in Sect.S1.1.2, is that algorithm B showed best performance on this data set. In Fig.S2. we show the LMI between the reconstructed hierarchy and the exact hierarchy as a function of the z-score threshold z^*. Although the obtained curve is rather flat in most of the examined region, setting the threshold to $z^* = 10$ in general seems as a good choice: below $z^* = 5$ the quality drops down, whereas no significant increase can be observed in $I_{\rm lin}$ between $z^* = 10$ and $z^* = 20$. By choosing $z^* = 10$, we ensure good quality, and also avoid throwing away too many connections.

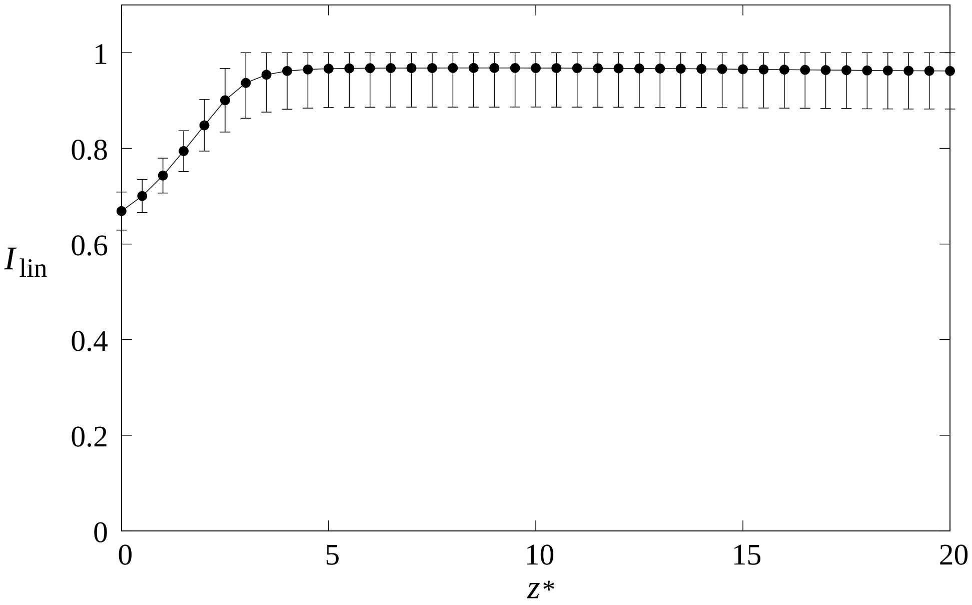

Figure S2. Optimizing the z-score threshold. We show the LMI as a function of the z-score threshold for the "hard" synthetic data set.

S2 Normalized mutual information

S2.1 NMI by partitioning of the tags

As mentioned in the main paper, a very important application of the concept of the NMI is given in community detection, where this measure can be used to quantify the similarity between partitions of the same network into communities by two alternative methods [1, 2]. The formula providing the NMI

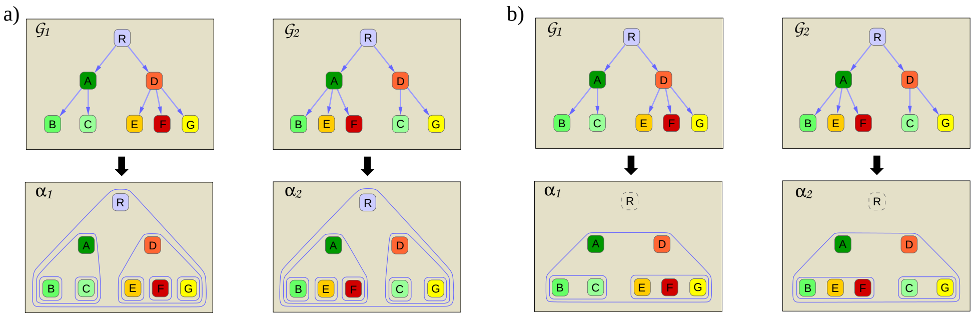

Figure S3. Mapping from hierarchies to communities. a) A simple intuitive mapping from the DAG to a communities of the tags in the DAG is given by nested sets, as shown here for $\mathcal{G}_1$ and $\mathcal{G}_2$, resulting in partitions α_1 and α_2. b) If we use instead communities given by the union of all descendants from non-leaf tags, (always excluding the given tag itself), the NMI given by (S1) becomes equivalent to the NMI defined for hierarchies in Eq.(1) in the main paper.

between community partitions α and β can be given as

$$I_{\alpha,\beta} = \frac{-2\sum_{i=1}^{C_\alpha}\sum_{j=1}^{C_\beta} N_{ij}\ln\left(\frac{N_{ij}N}{N_i N_j}\right)}{\sum_{i=1}^{C_\alpha} N_i \ln\left(\frac{N_i}{N}\right) + \sum_{j=1}^{C_\beta} N_j \ln\left(\frac{N_j}{N}\right)}, \tag{S1}$$

where C_α and C_β denote the number of communities in the two partitions, N_i and N_j stand for the number of nodes in communities i and j respectively, with N_{ij} giving the number of common nodes in i and j, and finally, N denoting the total number of nodes in the network. This measure can be used e.g., when judging the quality of a community finding method run on a benchmark for which the ground truth communities are known.

Meanwhile, (S1) is in complete analogy with our definition of the NMI for a pair of hierarchies, (Eq.(1) in the main paper): if we convert the hierarchies to be compared into community partitions in an appropriate way, the two measures become equivalent. Probably the most natural idea for a mapping from a DAG to communities of the tags in the DAG is turning the original "order" hierarchy represented by the DAG into a "containment" hierarchy of nested sets, as shown in Fig.S3a., (with each set corresponding to the union of tags in a given branch of the DAG). However, by applying (S1) to the partitions obtained in this way we obtain different results compared to Eq.(1) in the main paper, and the resulting similarity measure does not approach 0 even for independent random DAGs. The reason for this effect is that leafs in the DAG provide communities consisting of single nodes, and due to the relatively large number of leafs in a general DAG, we always obtain a non vanishing portion of exactly matching communities.

The mapping from a DAG to communities providing results equivalent to our NMI definition is obtained by associating with every tag in the DAG the union of its descendants, excluding the tag itself, (see Fig.S3b for illustration). This way the leafs appear only in the communities corresponding to their ancestors, thus, the emergence of a large number of communities with only a single member is avoided.

S2.2 Gene Ontology DAG

In Fig.1. in the main paper we have examined the behavior of the NMI between a binary tree and its randomized counterpart as a function of the fraction of rewired links. Here we show similar results

obtained for the exact hierarchy of our protein data set, obtained from the Gene Ontology [3]. In

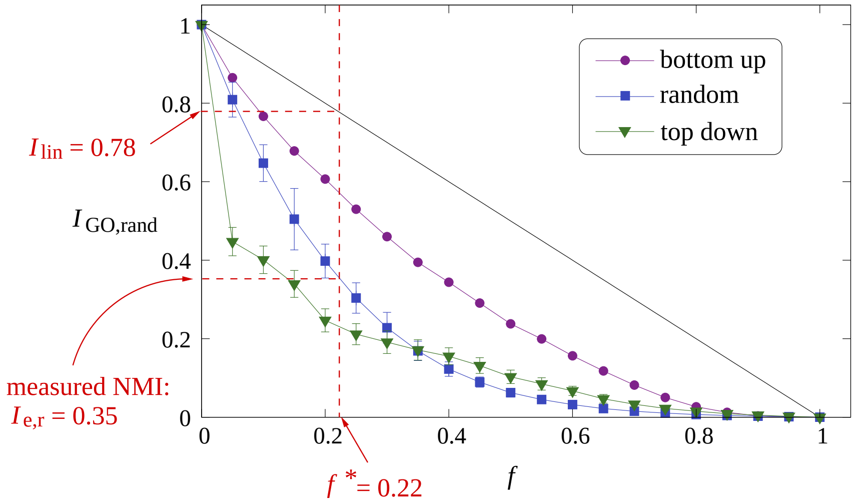

Figure S4. NMI decay for the exact hierarchy of protein tags and its randomized counterpart. We plotted I as given in Eq.(1) of the main paper as a function of the randomly rewired links, f. The three different curves correspond to rewiring the links in reverse order according to their position in the hierarchy (purple circles), rewiring in random order (blue squares) and rewiring in the order of the position in the hierarchy (green triangles). The red lines illustrate the calculation of the linearized mutual information for the reconstruction result obtained from algorithm A.

Fig.S4. the NMI defined in Eq.(1) of the main paper is shown for the exact hierarchy and its randomized counterpart as a function of the randomly rewired links, f. The three different curves correspond to three different orders in which the links were chosen for the rewiring: in case of the purple curve we started the rewiring with links pointing to leafs, and continued in reverse order according to the hierarchy, in case of the blue curve, the links were chosen in random order, while in case of the green curve, we started the rewiring at the top of the hierarchy, and continued in the order according to the hierarchy. Similarly to Fig.1. in the main paper, all three curves decay to 0 as $f \to 1$, thus, the similarity becomes 0 when the compared DAGs become independent. However, the behavior in the small and medium f regime is rather different: the green curve drops below $I_{\rm GO,rand} = 0.5$ already at $f = 0.05$, while the blue curve shows a moderate decrease and the purple curve decays even more mildly. Similarly to Fig.1. in the main paper, this justifies our statement that the NMI is sensitive also to the position of the links in the hierarchy: rewiring links high in the hierarchy has a larger effect on the similarity compared to rewiring links close to the leafs. Interesting, in the medium f^* regime a crossover can be observed between the green- and the blue curve. The possible explanation for this effect lies in the non-trivial, nor random, nor regular structure of the original DAG.

The red lines in Fig.S4. demonstrate the calculation of the linearized mutual information for the results obtained from algorithm A: The obtained NMI value of $I_{\rm e,r} = 0.37$ between the output of the algorithm and the exact DAG is projected to the f axis using the blue curve, resulting in $f^* = 0.22$. The linearized mutual information, $I_{\rm lin}$ is given by $1 - f^*$, resulting $I_{\rm lin} = 0.78$.

S3 Further results on Flickr and IMDb

S3.1 Additional samples from the Flickr hierarchy

The exact hierarchy between the tags appearing in Flickr is not known, thus, the quality of the extracted hierarchy can be judged only by "eye", i.e., by looking at smaller subgraphs, whether they make sense or not. In Fig.3. of the main paper we have already shown a part of the branch under "reptile" in the hierarchy obtained from algorithm B. Here we show further examples in the same manner. In Fig.S5. we depict a part of the hierarchy under the tag "winter", with very reasonable descendants like "snow", "ski", "cold", "ice", etc. Similarly, in Fig.S6. we show a part of the descendants of "rodent", displaying again a rather meaningful hierarchy.

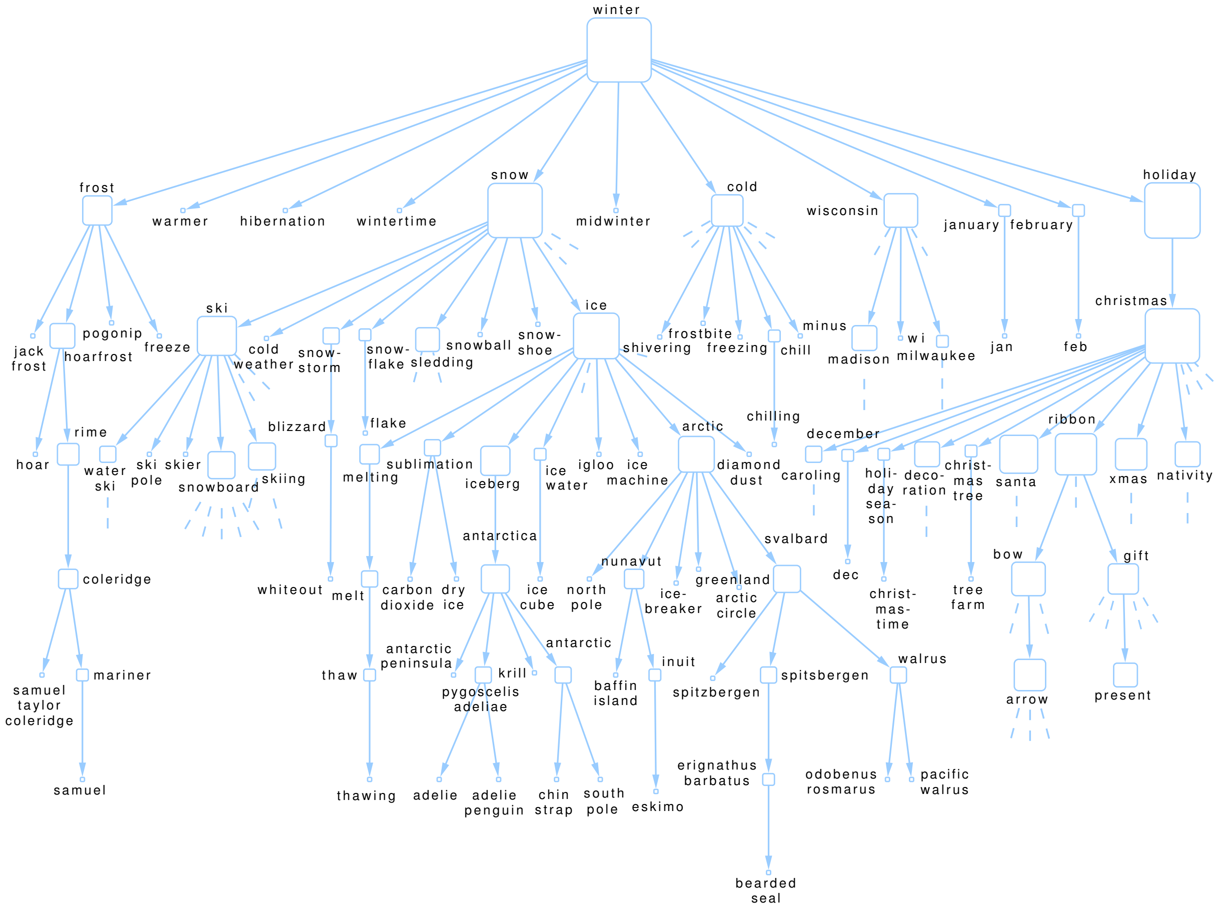

Figure S5. Partial subgraph of the descendants of "winter" in the hierarchy between Flickr tags obtained from algorithm B. Stubs (in dashed line) signal further descendants not shown in the figure, and the size of the nodes indicate the total number of descendants.

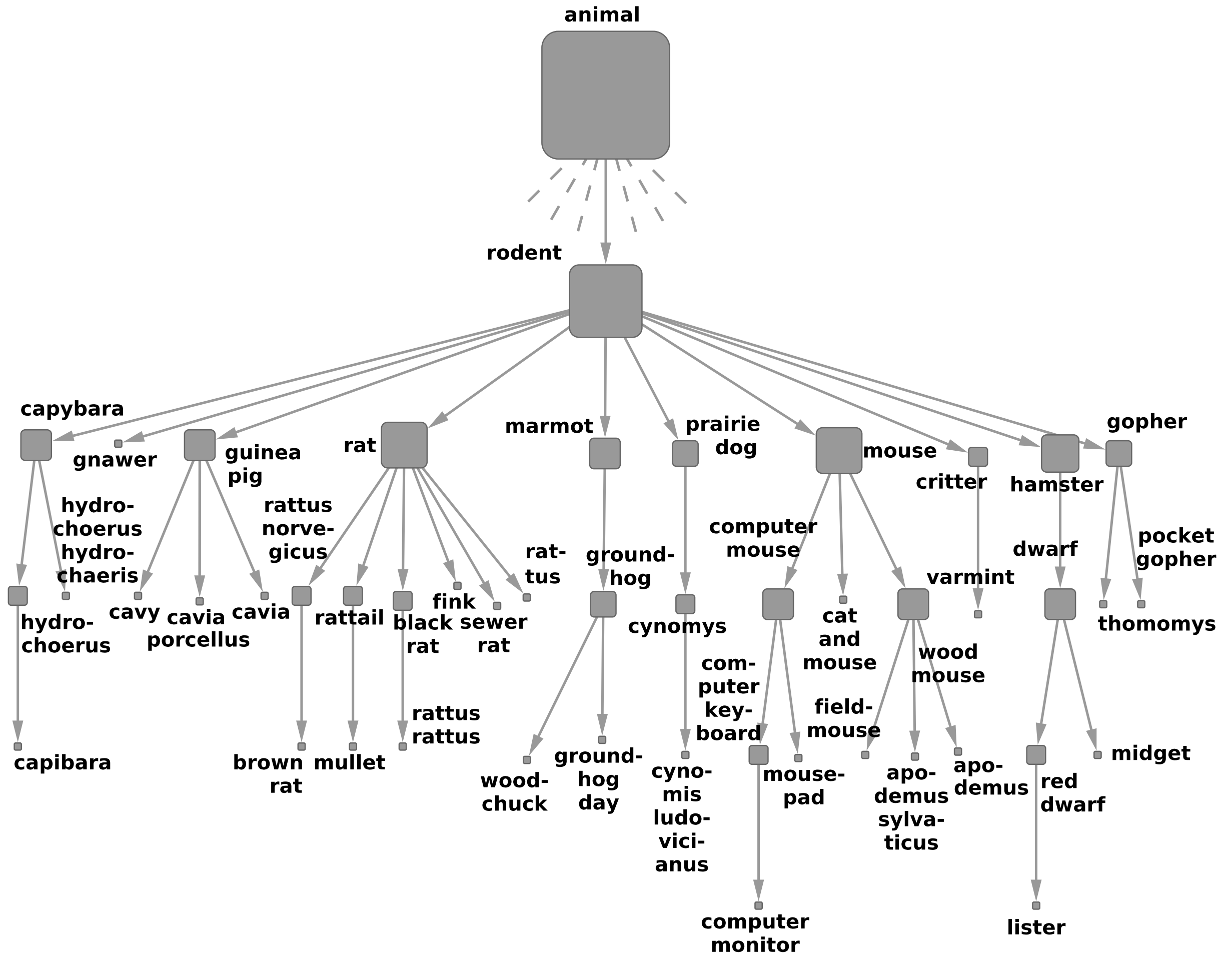

Figure S6. A part of the descendants of "rodent" in the hierarchy between Flickr tags. Similarly to Fig.S5., the overall hierarchy behind the subgraph shown here was obtained from algorithm B. Stubs (in dashed line) signal further descendants not shown in the figure, and the size of the nodes indicate the total number of descendants.

S3.2 Samples from the hierarchies extracted with the other methods

For comparison with Figs.3-4. in the main paper, here in Figs.S7-S12. we show the corresponding parts from the hierarchies extracted with algorithm A, the method by P. Heymann & H. Garcia-Molina and the algorithm by P. Schmitz. Since the overall structure of the hierarchies is varying over the different algorithms, naturally, the set of tags appearing in these figures is somewhat different compared to Figs.3-4. in the main paper. I.e., tags in direct ancestor-descendant relation according to algorithm B can be classified into different branches by an other algorithm or siblings may become unrelated etc. in the output of another method. Therefore, our strategy when preparing Figs.S7-S12. was to choose the largest branch, containing the most common tags with Figs.3-4. in the main paper.

In Figs.S7-S9. we show samples corresponding to Fig.3. in the main paper, obtained from the hierar-

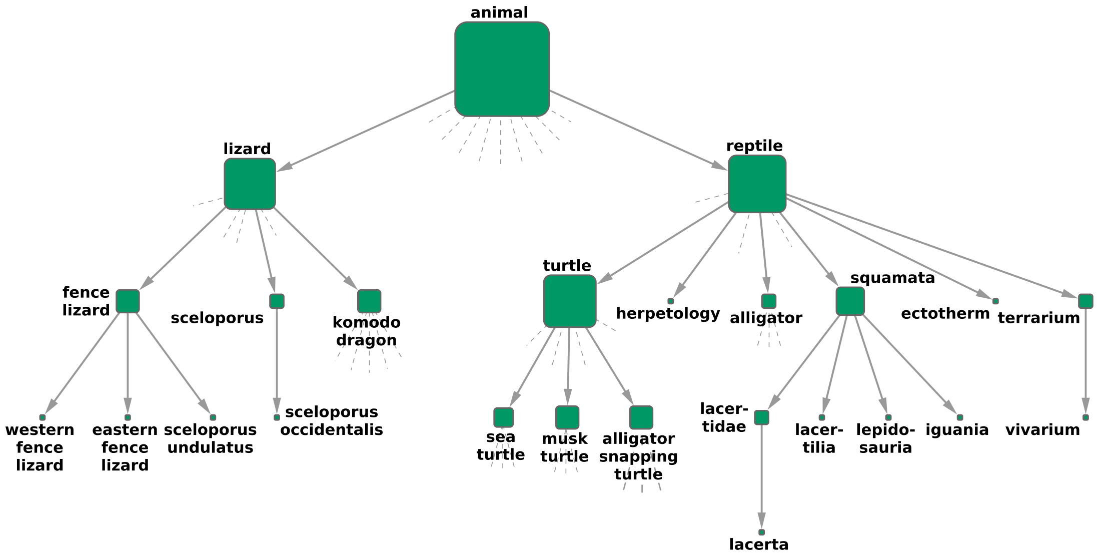

Figure S7. A part of the descendants of "reptile" and "lizard" in the hierarchy between Flickr tags obtained with algorithm A. Stubs (in dashed line) signal further descendants not shown in the figure, and the size of the nodes indicate the total number of descendants.

chies extracted for the Flickr tags. Interestingly, in case of algorithm A, (Fig.S7.), the tag "lizard" and "reptile" are classified into different branches. Meanwhile, in the subgraph obtained from the algorithm by P. Heymann & H. Garcia-Molina, (Fig.S8), the tag "snake" has been chosen to be the direct ancestor of "reptile". Apart from that, the hierarchy of the tags is rather similar to that shown in Fig.3. in the main paper. In case of the algorithm by P. Schmitz, the obtained result was actually composed of many distinct small hierarchies, with the tags given in Fig.3. in the main paper spreading over a large number of different components. Thus, we included a larger set of these small hierarchies in Fig.S9. instead of a single larger subgraph as in Figs.S7-S8.

In Figs.S10-S12. we show samples from the hierarchies obtained for the IMDb tags. In case of algorithm A, (Fig.S10.), we display the branch under "blood", as most of its descendants appear also on Fig.4 in the main paper, while the tag "murder" is missing from the figure, since it was sorted into a different branch. The subgraph shown for the method by P. Heymann & H. Garcia-Molina, (Fig.S11.), has similar features compared to Fig.4 in the main paper, however, the direct ancestor-descendant relation between "murder" and "death" has been reversed. Finally, the results for the algorithm by P. Schmitz are again very dispersed, thus, we included more than one small subgraph in Fig.S12.

S4 Synthetic benchmark

S4.1 Pseudo code

In Algorithm S4. we briefly sketch the pseudo code of the preparation of the synthetic tagged data in our benchmark system. As explained in the main paper, the basic idea is to use a random walk process on the pre-defined hierarchy for ensuring the higher frequency of co-occurrences between more closely related tags. Beside the hierarchy between the tags, the following parameters are also assumed to be

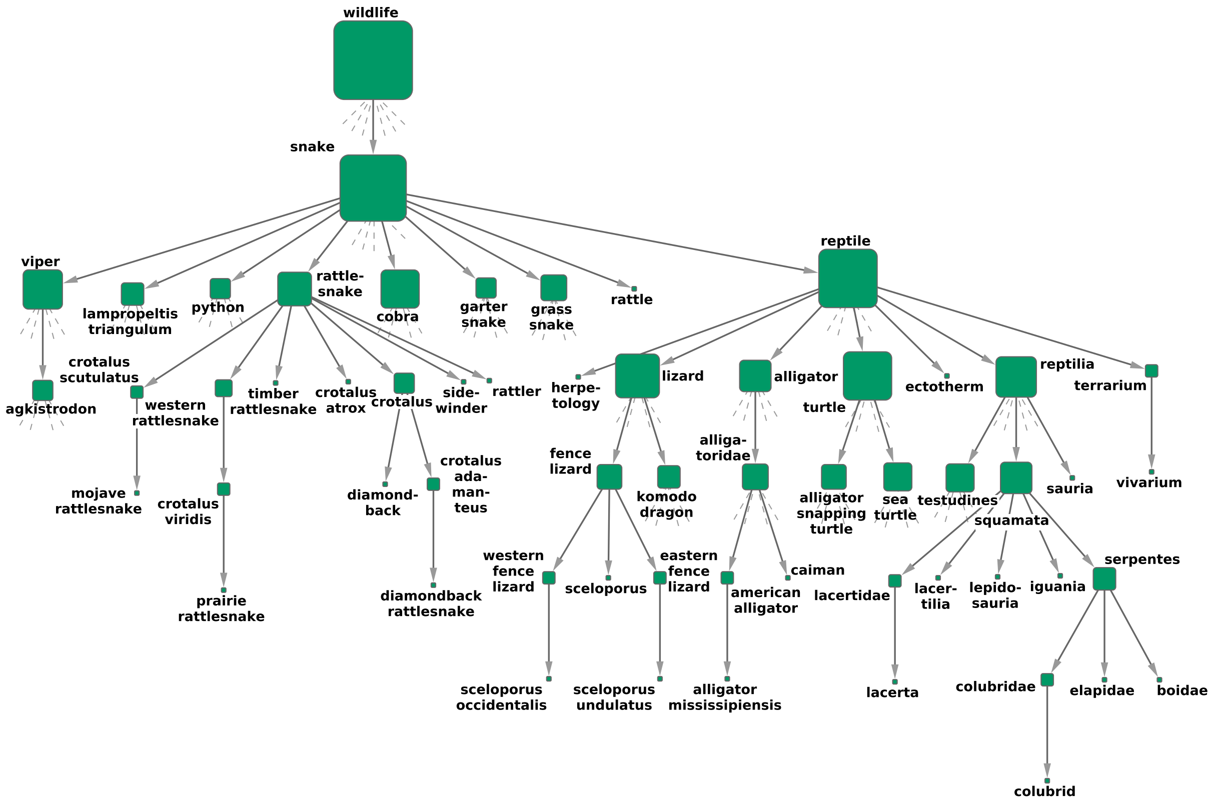

Figure S8. A part of the descendants of "snake" in the hierarchy between Flickr tags obtained with the method by P. Heymann & H. Garcia-Molina. Stubs (in dashed line) signal further descendants not shown in the figure, and the size of the nodes indicate the total number of descendants.

pre-defined: the number of virtual objects to be generated, the frequency distribution of the tags, the distribution of the number of tags on the objects and the distribution of the random walk lengths.

S4.2 Further tests based on the "easy" parameter settings

In the main paper we have shown that when the frequency of tags is decreasing linearly as a function of the depth in the hierarchy, the synthetic benchmark becomes "easy", and an almost perfect reconstruction becomes possible. As an illustration, in Fig.S13a-b we show parts from the exact DAGs, (binary trees of 1023 tags), used for testing algorithm A and algorithm B, respectively. In Fig.S13c we display the corresponding subgraph from the hierarchy obtained from algorithm A. The result is quite good, where the majority of the links are exactly matching, (colored green), while the rest are acceptable (shown in orange). However in case of algorithm B the chosen part of the reconstruction is perfect, as shown in Fig.S13d, with only exactly matching links.

According to the results discussed in the main paper, when the tag frequencies are independent of the position in the hierarchy and have a power-law distribution, the benchmark becomes hard. Here we examine the effect of changes in the other parameters of the benchmark. First, our starting point is the

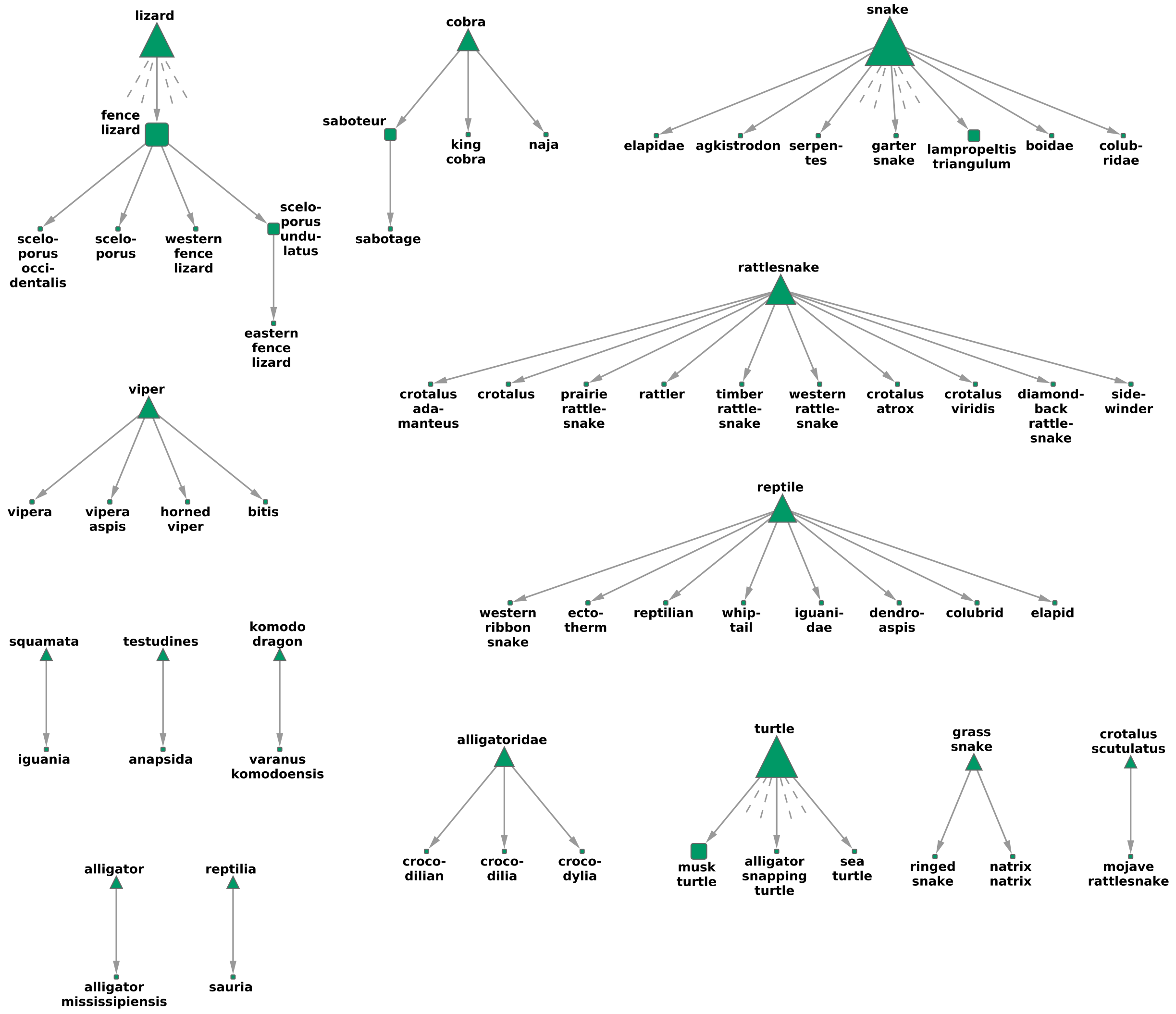

Figure S9. Samples from the small hierarchies between Flickr tags obtained with the algorithm by P. Schmitz. Triangular shaped nodes represent local roots. These were chosen from tags appearing in Fig.3. in the main paper.

"easy" parameter setting, while the results obtained for the "hard" parameter setting are discussed in Sect.S4.3. As mentioned in the main paper, the most important feature of the "easy" parameter settings is that the frequency of the tags is decreasing linearly as a function of the level depth in the hierarchy. The other parameters were set as follows: an average number of 3 co-occurring tags were generated on altogether 2,000,000 hypothetical objects, with random walk probability of $p_{\mathrm{RW}} = 0.5$ and random walk lengths chosen from a uniform distribution between 1 and 3, (the results are shown in Table 2. in the main paper). First we study the effect of changing the length of the random walks. In Table S1. we show the results when we decrease the random walk length to only a single step: according to the listed

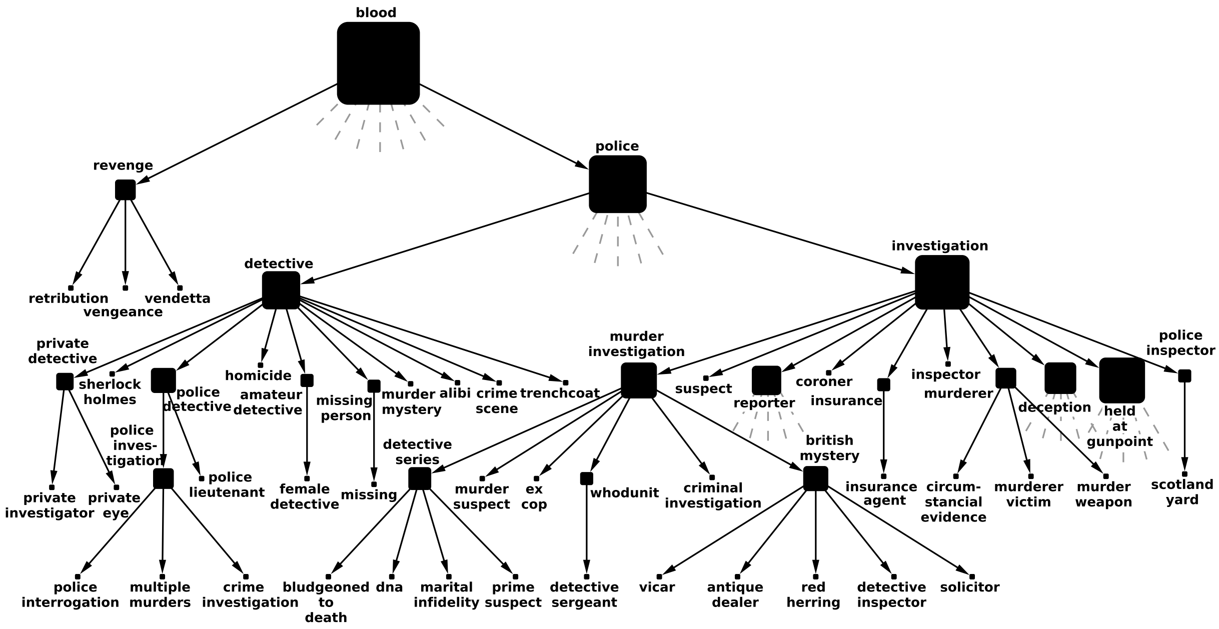

Figure S10. A part of the descendants of "blood" in the hierarchy between IMDb tags obtained with algorithm A. Stubs (in dashed line) signal further descendants not shown in the figure, and the size of the nodes indicate the total number of descendants.

Algorithm S4 Generating synthetic data based on random walk

---

for all virtual objects **do**
    draw tag t_1 at random according to the tag frequency distribution
    assign t_1 to the virtual object
    draw number of tags n_T at random from the distribution of the number of tags on the objects
    for all i=2, i <= n_T **do**
        if random number r < p_{RW} **then**
            draw random walk length l_{RW} at random from the random walk length distribution
            set tag $t_i = t_1$
            for all j=1, j <= l_{RW} **do**
                random walk on the pre-defined hierarchy, ignoring the link directions:
                new tag t_j := random neighbor of t_i
                set $t_i = t_j$
            end for
            assign t_i to the virtual object
        else
            draw tag t_i at random according to the tag frequency distribution
            assign t_i to the virtual object
        end if
    end for
end for

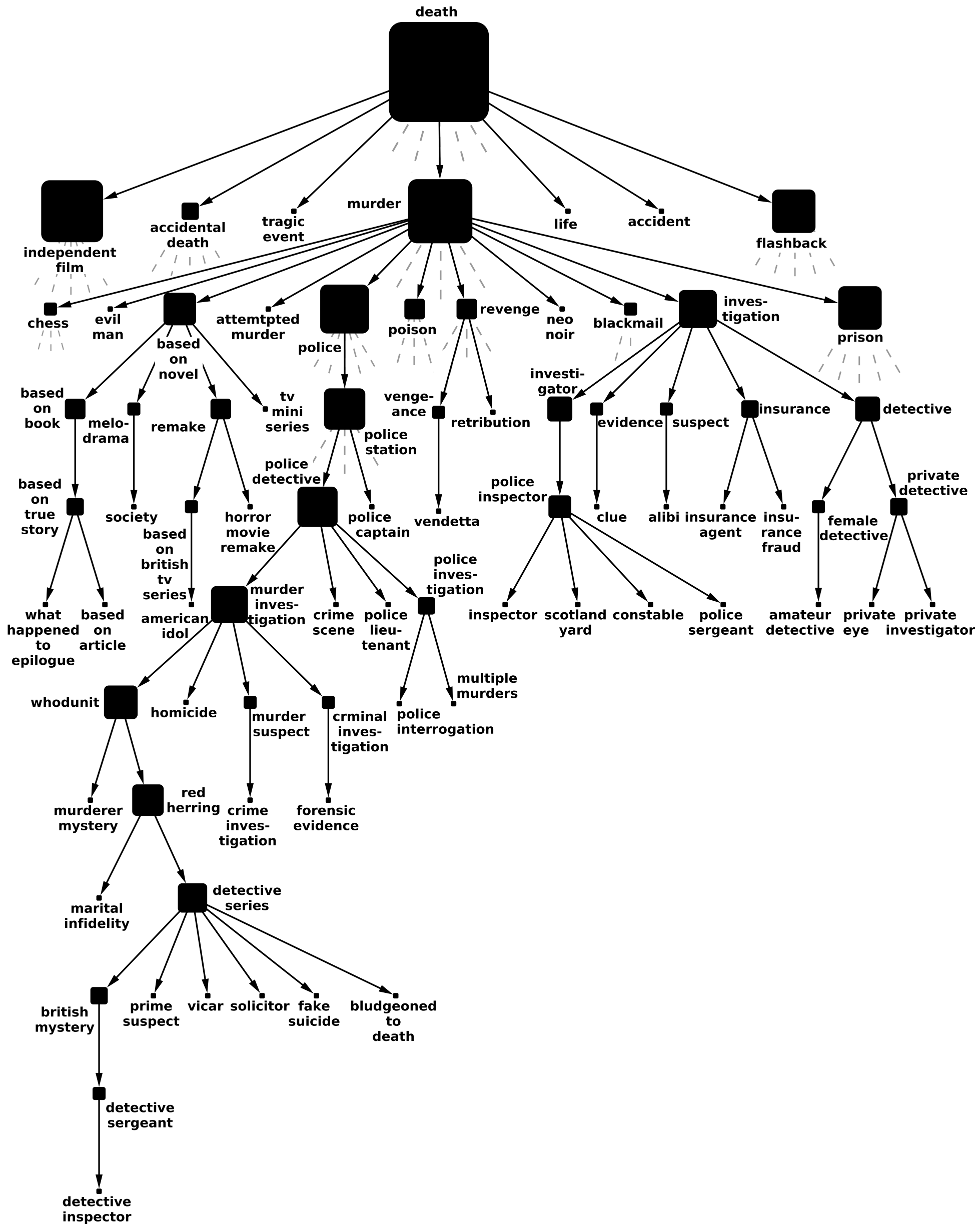

Figure S11. A part of the descendants of "murder" in the hierarchy between IMDb tags obtained with the method by P. Heymann & H. Garcia-Molina. Stubs (in dashed line) signal further descendants not shown in the figure, and the size of the nodes indicate the total number of descendants.

measures, the quality of the reconstruction for Algorithm B, the method by P. Heymann & H. Garcia-Molina and the algorithm by P. Schmitz remain exactly or almost exactly the same. In case of Algorithm

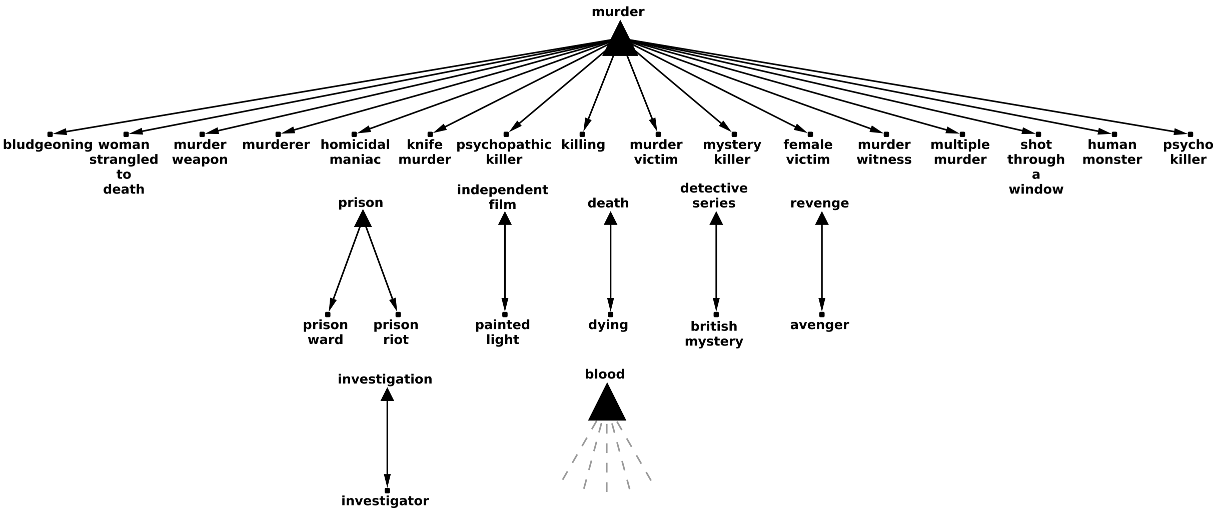

Figure S12. Samples from the small hierarchies between IMDb tags obtained with the algorithm by P. Schmitz Triangular shaped nodes represent local roots. These were chosen from tags appearing in Fig.4. in the main paper.

Table S1. Quality measures of the reconstructed hierarchies with random walk length of 1 step.

	$r_{\rm E}$	$r_{\rm A}$	$r_{\rm I}$	$r_{\rm U}$	$r_{\rm M}$	$I_{\rm e,r}$	$I_{\rm lin}$
algorithm A	68%	95%	0%	5%	0%	47%	86%
algorithm B	100%	100%	0%	0%	0%	100%	100%
P. Heymann & H. Garcia-Molina	99%	99%	1%	0%	0%	92%	99%
P. Schmitz	0%	0%	0%	0%	100%	0%	0%

The setting of the other parameters were exactly the same as in case of the “easy” synthetic data set discussed in the main paper.

A the quality indicators are somewhat lower compared to Table 2. in the main text, however, solely $I_{\rm e,r}$ is changed significantly. In Table S2. we show the results when the length of the random walks was chosen from a uniform distribution between 1 and 5, and the other parameters of the data set were left the same. Again, algorithm B, the method by P. Heymann & H. Garcia-Molina and the algorithm by P. Schmitz produce the same (or almost the same) results as presented in Table 2. of the main text. The results from algorithm A are now better compared to the original settings, reaching almost the same quality as algorithm A. In conclusion, the change in the length of the random walks has only a negligible effect for three out of the four methods studied here, and a mild effect on the results from the fourth one.

Next, we examine the effect of reducing the number of generated virtual objects. In Table S3. we show the results obtained when we generated only 200,000 virtual objects instead of 2,000,000, (and otherwise used the same parameters as in case of the “easy” synthetic data set). Not surprisingly, the quality of the methods show a slight decrease, as the hierarchy has to be reconstructed based on less information. However, the effect is only minor. When reducing the number of objects further down to 50,000, the drop in the quality measures becomes more pronounced, as presented in Table S4. Interestingly, the algorithm

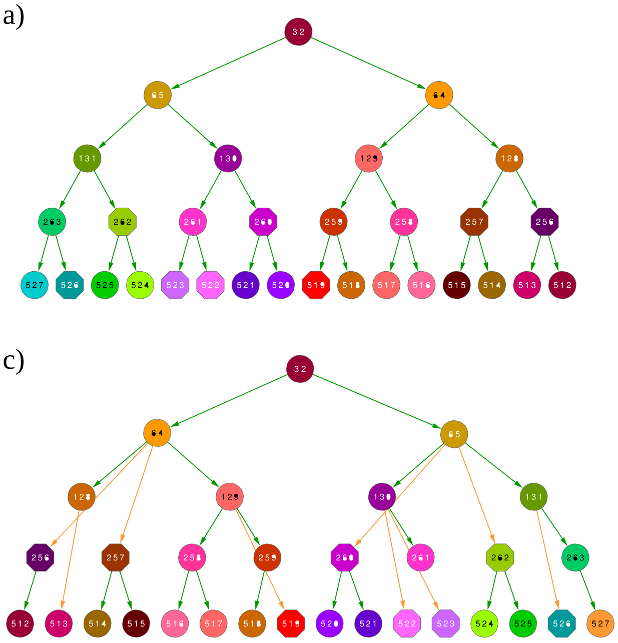

Figure S13. Comparison between the exact hierarchy and the reconstructed hierarchy in case of the "easy" computer generated benchmark. a) A subgraph from the exact hierarchy for testing algorithm A. b) A subgraph from the exact hierarchy for testing algorithm B. c) The subgraph corresponding to a) in the result obtained from algorithm A. Exactly matching links are shown in green, acceptable links are colored orange. d) The subgraph corresponding to b) in the result obtained from algorithm B, showing a perfect match.

Table S2. Quality measures of the reconstructed hierarchies with maximum random walk length of 5 step.

	r_{E}	r_{A}	r_{I}	r_{U}	r_{M}	$I_{\mathrm{e,r}}$	I_{lin}
algorithm A	95%	100%	0%	0%	0%	99%	100%
algorithm B	100%	100%	0%	0%	0%	100%	100%
P. Heymann & H. Garcia-Molina	99%	99%	0%	0%	0%	93%	99%
P. Schmitz	0%	0%	0%	0%	100%	0%	0%

The setting of the other parameters were exactly the same as in case of the "easy" synthetic data set discussed in the main paper.

by P. Schmitz shows a different behavior, with a slight increase in quality. As mentioned in the main paper, the study of the reasons for the outlying behavior of this algorithm on the synthetic data is out of the scope of present work.

Finally, in Table S5. we show the quality measures obtained when the random walk probability was reduced from $p_{\mathrm{RW}} = 0.5$ to $p_{\mathrm{RW}} = 0.1$, (and the other parameters were the same as in case of the "easy" synthetic data set). Similarly to the case of reducing the number of objects, this provides a more difficult

Table S3. Quality measures of the reconstructed hierarchies with 200,000 hypothetical objects

	r_E	r_A	r_I	r_U	r_M	$I_{e,r}$	I_{lin}
algorithm A	67%	97%	1%	2%	0%	86%	98%
algorithm B	98%	98%	2%	0%	0%	100%	100%
P. Heymann & H. Garcia-Molina	98%	98%	2%	0%	0%	93%	99%
P. Schmitz	0%	0%	0%	0%	100%	0%	0%

The setting of the other parameters were exactly the same as in case of the "easy" synthetic data set discussed in the main paper.

Table S4. Quality measures of the reconstructed hierarchies with 50,000 hypothetical objects

	r_E	r_A	r_I	r_U	r_M	$I_{e,r}$	I_{lin}
algorithm A	57%	89%	3%	8%	0%	75%	95%
algorithm B	70%	81%	13%	6%	0%	76%	95%
P. Heymann & H. Garcia-Molina	87%	91%	5%	4%	0%	94%	99%
P. Schmitz	2%	3%	0%	0%	97%	1%	11%

The setting of the other parameters were exactly the same as in case of the "easy" synthetic data set discussed in the main paper.

Table S5. Quality measures of the reconstructed hierarchies with random walk probability $p_{RW} = 0.1$.

	r_E	r_A	r_I	r_U	r_M	$I_{e,r}$	I_{lin}
algorithm A	1%	61%	0%	39%	0%	0%	1%
algorithm B	89%	90%	8%	2%	0%	64%	92%
P. Heymann & H. Garcia-Molina	88%	99%	1%	0%	0%	68%	93%
P. Schmitz	0%	0%	0%	0%	100%	0%	0%

The setting of the other parameters were exactly the same as in case of the "easy" synthetic data set discussed in the main paper.

task for the tag hierarchy extracting algorithms, as most of the tags are chosen at random on the objects. Accordingly, the quality measures are decreased when compared to the results shown in Table 2. of the main text. This effect is quite significant in case of algorithm A, while its less pronounced for algorithm B and the method by P. Heymann and H. Garcia-Molina.

S4.3 Further tests based on the "hard" parameter settings

In similar fashion to Sect.S4.2, here we examine the effects of changing the parameters when we start from the "hard" parameter setting. As mentioned in the main paper, the main feature making this choice of parameters "hard" is that the frequency of tags is independent of the level depth in the hierarchy. Otherwise, the parameters of the data set discussed in Table 3. of the main text were the following: an average number of 3 co-occurring tags were generated on altogether 2,000,000 hypothetical objects, with

random walk probability of $p_{\mathrm{RW}} = 0.5$ and random walk lengths chosen from a uniform distribution between 1 and 3. Starting from this parameter setting, in Table S6. we show the results obtained when the random walk length is reduced to 1. For all 4 methods, we can observe a slight increase in the quality, however, no significant changes have occurred when comparing to Table 3. in the main text.

Table S6. Quality measures of the reconstructed hierarchies with random walk length of 1 step.

	r_{E}	r_{A}	r_{I}	r_{U}	r_{M}	$I_{\mathrm{e,r}}$	I_{lin}
algorithm A	40%	40%	17%	43%	0%	21%	70%
algorithm B	92%	93%	5%	2%	0%	84%	97%
P. Heymann & H. Garcia-Molina	51%	55%	30%	15%	0%	28%	76%
P. Schmitz	4%	4%	0%	5%	91%	2%	18%

The setting of the other parameters were exactly the same as in case of the “hard” synthetic data discussed in the main paper.

In Table S7. we show the results when the length of the random walks was chosen from a uniform distribution between 1 and 5, and the other parameters of the data set were left the same as in case of Table 3. in the main text. Interestingly, this time the quality measures have been lowered slightly, nevertheless, no significant change can be observed. In a similar fashion to Sect.S4.2, our conclusion is that the length of the random walk has no significant effect on the quality of the examined algorithms.

Table S7. Quality measures of the reconstructed hierarchies with maximum random walk length of 5 step.

	r_{E}	r_{A}	r_{I}	r_{U}	r_{M}	$I_{\mathrm{e,r}}$	I_{lin}
algorithm A	28%	36%	29%	35%	0%	18%	66%
algorithm B	85%	88%	10%	2%	0%	81%	96%
P. Heymann & H. Garcia-Molina	46%	52%	34%	14%	0%	28%	76%
P. Schmitz	1%	1%	1%	4%	94%	1%	4%

The setting of the other parameters were exactly the same as in case of the “hard” synthetic data set discussed in the main paper.

We continue our experiments by changing the number of virtual objects in the preparation of the data set. In Table S8. we show the results obtained when we generated only 200,000 virtual objects instead of 2,000,000, (and otherwise used the same parameters as in case of the “hard” synthetic data set). The quality measures for algorithm A, the method by P. Heymann & H. Garcia-Molina and the algorithm by P. Schmitz remained almost the same, while the marks for algorithm B have been slightly reduced, (however, algorithm B is still far the best method on this data set). In Table S9. we show the results obtained when the number of hypothetical objects were further reduced to 50,000. In this case the quality of algorithm A, the method by P. Heymann & H. Garcia-Molina and the algorithm by P. Schmitz has slightly dropped, when compared to Table 3. in the main paper. The decrease in the quality is more pronounced in case of algorithm B, however, its results are still much better than that of the others. In conclusion, the lowering of the number of virtual objects affects most the result from algorithm B, nevertheless its quality was always significantly higher compared to the other methods.

Finally, in Table S10. we examine the effects of lowering the random walk probability from $p_{\mathrm{RW}} = 0.5$ to $p_{\mathrm{RW}} = 0.1$,(while keeping the other parameters the same as in case of the “hard” synthetic data set). As mentioned in Sect.S4.2, this provides a more difficult task for the tag hierarchy extracting algorithms,

Table S8. Quality measures of the reconstructed hierarchies with 200,000 hypothetical objects

	r_E	r_A	r_I	r_U	r_M	$I_{e,r}$	I_{lin}
algorithm A	31%	36%	26%	38%	0%	18%	66%
algorithm B	80%	86%	12%	2%	0%	76%	95%
P. Heymann & H. Garcia-Molina	48%	54%	32%	14%	0%	29%	76%
P. Schmitz	1%	2%	0%	4%	94%	1%	5%

The setting of the other parameters were exactly the same as in case of the "hard" synthetic data set discussed in the main paper.

Table S9. Quality measures of the reconstructed hierarchies with 50,000 hypothetical objects

	r_E	r_A	r_I	r_U	r_M	$I_{e,r}$	I_{lin}
algorithm A	29%	36%	26%	39%	0%	17%	65%
algorithm B	66%	74%	20%	6%	0%	55%	89%
P. Heymann & H. Garcia-Molina	46%	53%	33%	14%	0%	28%	76%
P. Schmitz	1%	2%	0%	5%	93%	1%	6%

The setting of the other parameters were exactly the same as in case of the "hard" synthetic data set discussed in the main paper.

as most of the tags are chosen at random on the objects. Accordingly, the quality measures are decreased when compared to the results shown in Table 3. of the main text. However, this effect is quite significant in case of algorithm A, while it is more mild for the method by P. Heymann & H. Garcia-Molina, and is even less pronounced in case of algorithm B. Our general conclusion regarding the robustness of the examined algorithms is that no significant differences could be observed in our experiments on the synthetic data sets. Algorithm B showed more sensitivity to the number of virtual objects compared to algorithm A and the method by P. Heymann & H. Garcia-Molina. In contrast, when reducing the random walk probability, algorithm B was found to be more robust compared to the other methods.

Table S10. Quality measures of the reconstructed hierarchies with random walk probability $p_{RW} = 0.1$.

	r_E	r_A	r_I	r_U	r_M	$I_{e,r}$	I_{lin}
algorithm A	10%	12%	14%	74%	0%	5%	33%
algorithm B	65%	71%	21%	8%	0%	61%	91%
P. Heymann & H. Garcia-Molina	35%	36%	34%	30%	0%	18%	66%
P. Schmitz	0%	0%	0%	7%	93%	0%	0%

The setting of the other parameters were exactly the same as in case of the "hard" synthetic data set discussed in the main paper.

References

1. Danon L, Díaz-Guilera A, Duch J, Arenas A (2005) Comparing community structure identification. J Stat Mech .

2. Lancichinetti A, Fortunato S, Kertész J (2009) Detecting the overlapping and hierarchical community structure in complex networks. New J Phys 11: 033015.

3. Consortium TGO (2000) Gene ontology: tool for the unification of biology. Nature Genetics 25: 25–29.
